# Structural Insights into RNA Polymerase Recognition and Essential Function of *Myxococcus xanthus* CdnL

**DOI:** 10.1371/journal.pone.0108946

**Published:** 2014-10-01

**Authors:** Aránzazu Gallego-García, Yasmina Mirassou, Diana García-Moreno, Montserrat Elías-Arnanz, María Angeles Jiménez, S. Padmanabhan

**Affiliations:** 1 Departamento de Genética y Microbiología, Área de Genética (Unidad Asociada al IQFR-CSIC), Facultad de Biología, Universidad de Murcia, Murcia, Spain; 2 Instituto de Química Física ‘Rocasolano’, Consejo Superior de Investigaciones Científicas (IQFR-CSIC), Madrid, Spain; Spanish National Cancer Center, Spain

## Abstract

CdnL and CarD are two functionally distinct members of the CarD_CdnL_TRCF family of bacterial RNA polymerase (RNAP)-interacting proteins, which co-exist in *Myxococcus xanthus*. While CarD, found exclusively in myxobacteria, has been implicated in the activity of various extracytoplasmic function (ECF) σ-factors, the function and mode of action of the essential CdnL, whose homologs are widespread among bacteria, remain to be elucidated in *M. xanthus.* Here, we report the NMR solution structure of CdnL and present a structure-based mutational analysis of its function. An N-terminal five-stranded β-sheet Tudor-like module in the two-domain CdnL mediates binding to RNAP-β, and mutations that disrupt this interaction impair cell growth. The compact CdnL C-terminal domain consists of five α-helices folded as in some tetratricopeptide repeat-like protein-protein interaction domains, and contains a patch of solvent-exposed nonpolar and basic residues, among which a set of basic residues is shown to be crucial for CdnL function. We show that CdnL, but not its loss-of-function mutants, stabilizes formation of transcriptionally competent, open complexes by the primary σ^A^-RNAP holoenzyme at an rRNA promoter *in* *vitro*. Consistent with this, CdnL is present at rRNA promoters *in* *vivo.* Implication of CdnL in RNAP-σ^A^ activity and of CarD in ECF-σ function in *M. xanthus* exemplifies how two related members within a widespread bacterial protein family have evolved to enable distinct σ-dependent promoter activity.

## Introduction

A single multisubunit DNA-dependent RNA polymerase (RNAP) holoenzyme composed of a catalytically competent core of five subunits (α_2_ββ′ω) and a given σ subunit initiates transcription from specified promoters in bacteria [1]. Mechanisms regulating this crucial transcription initiation step enlist a variety of factors among which are proteins that directly bind to RNAP but not to DNA [1], [2]. One such well-studied factor is DksA, found in *E. coli* and many other bacteria, which targets promoters of genes encoding rRNA and ribosomal proteins, as well as those of many amino acid biosynthesis operons [2], [3]. Recent studies uncovered a widely distributed class of bacterial proteins that also function by interacting with RNAP, the large CarD_CdnL_TRCF family ([4]–[7]; PF02559 in the protein family database, http://pfam.sanger.ac.uk). This family is defined by CarDNt, the ∼180-residue N-terminal domain of the global transcriptional regulator CarD, which acts in light-induced carotenogenesis (hence the name CarD), starvation-induced development of multicellular fruiting bodies, and other processes in the Gram-negative soil bacterium *Myxococcus xanthus* [8], [9]. The family includes the RNAP-interacting domain (RID) of the transcription-repair coupling factor (TRCF), a large, widely conserved, multidomain bacterial protein that mediates transcription-coupled repair of DNA lesions encountered by the transcribing complex [10]–[13]. Both CarD and TRCF have domains that directly contact DNA. In CarD, this is an ∼140-residue intrinsically unfolded C-terminal domain that resembles eukaryotic high mobility group A (HMGA) proteins in its structural and DNA-binding properties [6], [7], [14], [15]. In TRCF, C-terminal domains mediate ATP-dependent DNA-binding [10]–[12]. CarDNt, indispensable for function, does not interact with DNA but with other proteins: itself, RNAP (specifically its β-subunit), and CarG, a zinc-associated transcriptional factor that also does not bind to DNA and is essential in every CarD-dependent process [4], [6], [7], [15], [16]. We recently discovered that CarD and CarG are required to activate transcription at target promoters of several distinct alternative, extracytoplasmic function σ (ECF-σ) factors, of which as many as ∼45 exist in *M. xanthus* [17].

A large class within the CarD_CdnL_TRCF family are standalone proteins with sequences similar to CarDNt but with no identifiable DNA-binding domain that we have named CdnL (for CarD N-terminal like) to distinguish them from CarD, since both proteins co-exist in *M. xanthus* [4]–[6]. *M. xanthus* CdnL, which localizes to the nucleoid *in* *vivo* and is essential for viability, is functionally distinct from CarD, and although both interact with RNAP-β, only CarD does so with CarG [4]. Unlike CarD, whose orthologs have thus far been identified only in myxobacteria [6], [15], [16], counterparts of the smaller CdnL are far more prevalent in bacteria and are increasingly identified due to the massive output of sequenced bacterial genomes [4]–[6], [9]. The mycobacterial CdnL homolog (hereafter MtCdnL, but described as CarD in other reports) is also vital for growth and interacts with RNAP-β [5]. It was originally reported to be a repressor of rRNA transcription, like DksA in *E. coli* [5], but this has now been revised to being an activator of rRNA transcription, and proposed to be a global regulator of transcription initiation at promoters recognized by RNAP holoenzyme with the major housekeeping σ (σ^A^) [18]. Whereas mycobacteria lack DksA, the latter co-exists with CdnL in *M. xanthus* and is also essential [4], [19], but their roles in rRNA transcription, if any, are unknown in this bacterium. Moreover, the simultaneous presence of CarD in *M. xanthus* and the shared ability of all three proteins to interact with, and so compete for, cellular RNAP, suggests a crosstalk that could have functional consequences. A knowledge of the molecular details of their various interactions is therefore necessary to understand the interplay between them and their modes of action.

The present study reports the two-domain architecture of *M. xanthus* CdnL and the NMR structures of each of these domains and of the full-length protein. We also describe our structure-based analysis of mutations that result in loss of the essential CdnL function and impair cell viability. These include mutations that disrupt the interaction with RNAP-β as well as those that leave this interaction intact. We present data that CdnL stabilizes open complex formation and stimulates transcription at an rRNA promoter by RNAP holoenzyme containing the major *M. xanthus* housekeeping σ^A^, and that the loss-of-function CdnL mutants lack this activity. Our results are discussed in the context of our data of the RNAP recognition domain of TtCdnL, the *T. thermophilus* CdnL homolog [20] and those from other groups on full-length TtCdnL and MtCdnL, both of which exist in bacteria lacking CarD as well as DksA [18], [21]–[23]. The involvement of CdnL in σ^A^-dependent rRNA promoter activity and of CarD in the action of several ECF-σ factors thus illustrates the evolution of two related members of an important bacterial protein family to regulate promoter activity dependent on different σ factors.

## Materials and Methods

### Strains, plasmids, growth conditions, and strain construction

Table S1 in File S1 lists strains and plasmids used in this study. *M. xanthus* was grown at 33°C in CTT (casitone Tris) medium that, as required, was supplemented with antibiotic (40 µg/ml kanamycin, Km; 10 µg/ml tetracycline, Tc) or 0.75 µM vitamin B_12_, or exposed to light from three 18-W fluorescent lamps (10 W/m^2^ intensity). Conditional gene expression in *M. xanthus* was carried out as described previously [4]. *E. coli* (strain DH5α for plasmid constructs, BL21-(DE3) for protein overexpression, and BTH101 (*cya* ^−^) for two-hybrid analysis) was grown in Luria-Bertani (LB) broth at 37°C. Intein and His-tagged proteins were overexpressed overnight at 18°C and 25°C, respectively, with 0.5 mM IPTG (isopropyl β-D-1-thiogalactopyranoside) in LB or, for [^13^C, ^15^N]-labeled proteins, in M9 or MOPS minimal medium containing 1 g/l ^15^NH_4_Cl and 2.5 g/l ^13^C_6_-glucose as the sole nitrogen and carbon sources [20], [24], [25]. Standard protocols and kits were used for plasmid constructs, all of which were verified by DNA sequencing. Plasmid pMR2873, with a Km^R^ marker for negative selection and *galK* for galactose sensitivity (Gal^S^) positive selection, was used in complementation analysis. In pMR2873, *cdnL* variants can be inserted into an XbaI site flanked on the 5′ and the 3′ ends, respectively, by ∼750 bp of the DNA segments upstream and downstream of *cdnL* in the genome for chromosomal integration. Site-directed mutants were obtained by overlap PCR, the QuikChange kit (Agilent), or gene synthesis (GenScript). CdnL, its homologs and RNAP-β segments were PCR-amplified from genomic DNA [4]. Strain MR1467 (Δ*cdnL* with *cdnL* conditionally expressed at a heterologous site) was electroporated with constructs (above) bearing *cdnL* or variants, selected for chromosomal integration of plasmids by homologous recombination at the endogenous *cdnL* locus, and analyzed for complementation as described before [4]. Stable protein expression was checked by immunoblot analysis of whole cell extracts using polyclonal anti-CdnL antibodies [4], [6], [15].

### Bacterial two-hybrid (BACTH) analysis and β-galactosidase activity

The *E. coli* BACTH system used is based on functional complementation of the T25 and T18 fragments of the *Bordetella pertussis* adenylate cyclase catalytic domain when two test proteins interact [26]. Coding regions of interest were PCR-amplified and cloned into the XbaI and BamHI sites of pKT25, pUT18 or pUT18C (Table S1 in File S1). Given pKT25-pUT18/pUT18C construct pairs were electroporated into *E. coli* BTH101 (*cya* ^−^), and pairs with one vector empty served as negative controls. Interaction was assessed from reporter *lacZ* activity, qualitatively from the blue colour developed on 40 µg/ml X-Gal (5-bromo-4-chloro-3-indolyl-β-D-galactoside) plates and quantitatively from the β-gal activity (in nmol of *o*-nitrophenyl β-D-galactoside hydrolysed/min/mg protein, reported as the mean and standard error of three or more independent experiments) of liquid cultures measured in a SpectraMax 340 microtitre plate reader (Molecular Devices), as described elsewhere [27].

### Protein purification and analysis

Purification of *M. xanthus* RNAP [28], and of H_6_- or intein-tagged CdnL and its fragments (including [^13^C, ^15^N]-labeled forms, with the tags removed by thrombin or intramolecular intein cleavage) has been described elsewhere [4], [20], [24], [25]. CdnL, its fragments or CarDNt do not bind to a phosphocellulose column [4], and passing CdnL samples through such a column in a final purification step ensured the elimination of impurities that could interfere in DNA-binding assays. Similar procedures were used for H_6_-TtCdnL purification and tag removal. Since TtCdnL binds to phosphocellulose, it was purified using MonoS ion-exchange (elutes between 0.2–0.3 M of a 0.1–1 M NaCl gradient) followed by size-exclusion chromatography. Protein identities were verified using N-terminal sequencing and mass spectrometry, and their concentrations were estimated using absorbance at 280 nm using ε_280_ (M^−1^cm^−1^) calculated from sequence (http://web.expasy.org/protparam/) or the BioRad protein assay kit. Size-exclusion analysis of purified proteins was done at room temperature with a Superdex-200 analytical HPLC column equilibrated with buffer (150 mM NaCl, 50 mM phosphate, pH 7.5, 2 mM β-mercaptoethanol) and calibrated as described previously [7]. The apparent molecular weight, *M*_r_, was estimated from the elution volume (*V*_e_) and eluted peak identity was verified using SDS–PAGE.

### Limited proteolysis

Pure protein (5 µg) in 120 µl of 100 mM NaCl, 50 mM Tris, pH 7.5, 1 mM β-mercaptoethanol was digested with subtilisin Carlsberg (Sigma-Aldrich) at 30°C (1∶100 w/w protein:protease). Aliquots of 20 µl were removed at 0, 15, 45, 60, 90, and 120 min, the protease inactivated with 1 µl each of 1 M phenylmethylsulfonyl fluoride (PMSF) and benzamidine, and analyzed in 15% SDS-PAGE gels with Coomassie Blue staining. A 60-min digest of an identical 40 µl sample was divided into two, and subjected to SDS-PAGE; with one sample, subtilisin-resistant bands identified by Coomasie Blue staining were excised from the gel and analyzed by MALDI-TOF mass spectrometry. The other sample was electrotransferred to Immobilon PSQ membrane (Millipore, MA), and the bands excised after staining with Coomasie Blue were subject to N-terminal sequencing.

### Circular dichroism (CD) spectroscopy

Far-UV CD spectra were recorded in a Pistar unit (Applied Photophysics, UK) calibrated with (+)-10-camphorsulfonic acid and coupled to a Peltier temperature control unit/Neslab RTE-70 water bath. Data were collected in 0.2 nm steps in the adaptive sampling mode at 25°C with 5–10 µM protein, 100 mM KF, 7.5 mM phosphate buffer (pH 7.5), 1 mm path length, 2 nm slit width, and averaged over three scans. Helix contents were estimated from [Θ]_222_, the mean residue ellipticity at 222 nm in degcm^2^dmol^−1^ using [Θ]_222_ = 895 for 0% helix and (−37,750) (1–3/N_r_) for 100% helix, N_r_ being the number of residues [29].

### NMR

NMR data were acquired in a Bruker AV-600 or AV-800 US2 spectrometers equipped with a z-gradient triple resonance cryoprobe using 0.5 or 0.2 mL samples of 0.5–1 mM protein in 100 mM NaCl/50 mM sodium phosphate buffer (pH 7.0, calibrated with a glass microelectrode and uncorrected for isotope effects)/0.05% NaN_3_ in 9∶1 v/v H_2_O/D_2_O or pure D_2_O. Probe temperatures were set using a methanol sample. Standard triple resonance NMR methods were used for data acquisition, processing, and ^1^H/^15^N/^13^C NMR chemical shift assignments (deposited at BioMagResBank; http://www.bmrb.wisc.edu/; [20], [24], [25]). Distance constraints, obtained from a 3D NOESY [^1^H-^13^C]-HSQC and two 2D [^1^H-^1^H]-NOESY spectra (mixing times of 80 ms and 150 ms, respectively) in H_2_O/D_2_O and/or D_2_O, and (φ, ψ) torsion angle constraints, from TALOS, were used as input in structure calculations using a standard iterative protocol of the program CYANA 2.1 [20], [30], [31]. Of the 100 conformers generated, 20 with the lowest target function values were energy minimized using AMBER9 (Case DA, Darden TA, Cheatham III TE, University of California, San Francisco, 2006). Constraints for CdnLNt and CdnLCt aided in guiding CYANA calculations of full-length CdnL. One-bond ^1^H-^15^N residual dipolar couplings were measured from the signal splitting in the ^15^N dimension in F1-coupled HSQC spectra recorded with a pulse sequence to separate the doublet into two sub-spectra in the F1 dimension [32]. Aliquots of 1 µL of *n*-octanol to 5% *n*-octyl-pentaethyleneglycol, C8E5 (w/v in 9∶1 v/v H_2_O/D_2_O) to a final molar ratio of 0.87∶1 C8E5:*n*-octanol were added to lyophilized [^15^N,^13^C] CdnL (1 mM, pH 7.0) and oriented sample anisotropy was verified from the 25 Hz doublet ^2^H splitting for solvent at 20°C [33]. Residual dipolar couplings (RDC) were obtained from the difference in splitting between spectra acquired for anisotropic (scalar and dipolar coupling contributions, ^1^J_NH_+^1^D_NH_) and isotropic (scalar coupling, ^1^J_NH_) conditions at 20°C and 28°C, respectively; and including RDC (11 and 9, respectively, in the N- and C-terminal domains) in the structure calculations significantly refined the CdnL ensemble. We used PROCHECK/NMR [34] to assess the quality of the final structures, MOLMOL [35] and PyMOL (Version 1.5.0.4 Schrödinger, LLC) for structural representations, and MOLMOL to calculate electrostatic surface potentials at 150 mM salt and default solute/solvent dielectric constant values. Heteronuclear [^15^N-^1^H] NOEs for backbone amides were estimated from the peak intensity ratios in [^1^H-^15^N] HSQC data obtained with and without NOE, recorded for [^13^C, ^15^N]-labeled NMR samples in H_2_O/D_2_O 9∶1 (v/v) at 800 MHz. For chemical shift perturbations, [^1^H-^15^N] or [^1^H-^13^C] HSQC spectra were recorded at 25°C after each addition of unlabeled Mxβ_19–148_ (0.4–5.0 equivalents) to 0.10–0.25 mM [^15^N, ^13^C] CdnL, CdnLNt or CdnlCt.

### Electrophoretic Mobility Shift Assays (EMSA) and *in* *vitro* run-off transcription

EMSA and *in* *vitro* transcription were carried out as described previously [28]. EMSA samples (20 µL) contained <1 nM ^32^P-5′-end radiolabeled double-stranded 130-bp P_B_, or 151-bp or 329-bp P_4*rrnD*_ DNA probes obtained by PCR (<13,000 cpm) and proteins at required concentrations in EMSA buffer (80 mM KCl, 25 mM Tris pH 8.0, 5 mM MgCl_2_, 1 mM dithiothreitol, 10% glycerol, 200 ng/µl bovine serum albumin) with or without 1 µg of poly[dG-dC] or, as required, poly[dI-dC] as nonspecific competitor. Samples were incubated for 30-min at 37°C. After adding 1 µg heparin and incubating for 5-min at 37°C, heparin-resistant open promoter complexes were electrophoresed (200 V, 1.5 h) in 4% nondenaturing PAGE gels in TBE buffer (45 mM Tris and boric acid, 1 mM EDTA) at 10°C. The gel was vacuum dried and analyzed by autoradiography. For *in* *vitro* transcription, 30 µL samples in EMSA buffer with unlabeled 329-bp P_4*rrnD*_ DNA probe, 20 units Protector RNase inhibitor (Roche), 130 nM RNAP, were incubated without or with 5 µM CdnL or its variants for 30 min at 37°C. Heparin was then added to 1 µg followed by 400 µM each ATP, UTP and GTP, and then 20 µM CTP, and 0.42 µM [α-^32^P] CTP to initiate transcription. The reaction after 15 min was quenched with 25 mM EDTA at 37°C. Free nucleotides were removed twice with ammonium acetate/ethanol precipitation, and the pellet was resuspended in 5 µl gel loading buffer II (Ambion) for electrophoresis in 8 M urea-6% PAGE gels and analyzed by autoradiography.

### Chromatin immunoprecipitation-quantitative PCR (ChIP-qPCR)

Cell cultures (50 mL) grown in CTT to mid-late exponential phase (OD_550_: 0.8) were cross-linked with 1% final concentration (v/v) formaldehyde (Merck) for 30 min at room temperature with shaking (100 rpm) and then quenched with glycine (2.5 ml of a 2.1 M stock). Cells were pelleted, washed thrice with phosphate-buffered saline and stored at −80°C until further use. The frozen pellet was thawed and resuspended in 200 µl ChIP lysis buffer A (20% sucrose, 50 mM NaCl, 10 mM EDTA 10 mM Tris pH 8, 1 mg/ml lysozyme) for 30 min at 37°C and cooled in ice. 800 µl ChIP lysis buffer B (150 mM NaCl, 1 mM EDTA, 50 mM HEPES-KOH pH 7.5, 1% Triton X-100, 0.1% deoxycholate, 0.1% SDS) with Complete protease inhibitor cocktail (Roche) was added, then sonicated using 12 cycles (30 s on, 30 s off) in a Bioruptor (Diagenode) to yield ∼0.5 kb long fragments and clarified by centrifugation. After keeping aside 20 µL of the supernatant as the input sample, the rest was added to 30 µl of polyclonal anti-CdnL antibodies [4] previously immobilized (≥4 hr incubation at 4°C and two washes with PBS containing 5 mg/ml BSA) on protein A magnetic Dynabeads (Life Technologies), and incubated overnight at 4°C with rotation. The beads were washed twice each with ChIP lysis buffer B, then this buffer with 0.5 M NaCl, and with wash buffer (250 mM LiCl, 10 mM Tris-HCl pH 8.0, 1 mM EDTA, 0.5% NP-40, 0.5% sodium deoxycholate). After a final wash with Tris-EDTA (TE) buffer, the beads were resuspended in 60 µL TE, 1% SDS, and incubated for 10 min at 65°C. From this, 40 µL together with 40 µL TE/1% SDS and 2.4 µl proteinase K (20 µg/µl) was incubated at 42°C for 2 h, followed by 65°C for 6 h, and DNA was isolated using the Roche High Pure PCR product Purification kit. The same cross-link reversal and DNA extraction protocols were used with the input sample. qPCR was performed in 0.1 ml MicroAMP FAST optical 48-well reaction plates and a StepOne qPCR apparatus (Applied Biosystems) using SYBR Green reaction mix (BioRad) and as primer pairs 5′-tgggcggcgctgaa-3′ and 5′-cagttcggcgtcttctgtca-3′ for P_4*rrnD*_, 5′-cccgctgccagagagatg-3′ and 5′-ttgtacacagaggtcccctcatg-3′ for P_B,_ and 5′- tcgcaaccccgtgacttt-3′and 5′-cgctcgcgcttctcaag-3′ for P_QRS_. An intragenic region between nucleotides 701–779 of the first gene of the P_B_-driven *M. xanthus carB* operon served as a non-promoter control region. Standard curves were obtained for each DNA region of interest and its corresponding primer pair with serially diluted input DNA sample. Signal enrichment at each promoter is expressed as the ratio of the promoter-specific to intragenic signals of the ChIP fractions relative to the values obtained with the input sample, and reported as the mean and standard error from three independent experiments.

## Results

### CdnL domain architecture and interactions

**Figure 1:**
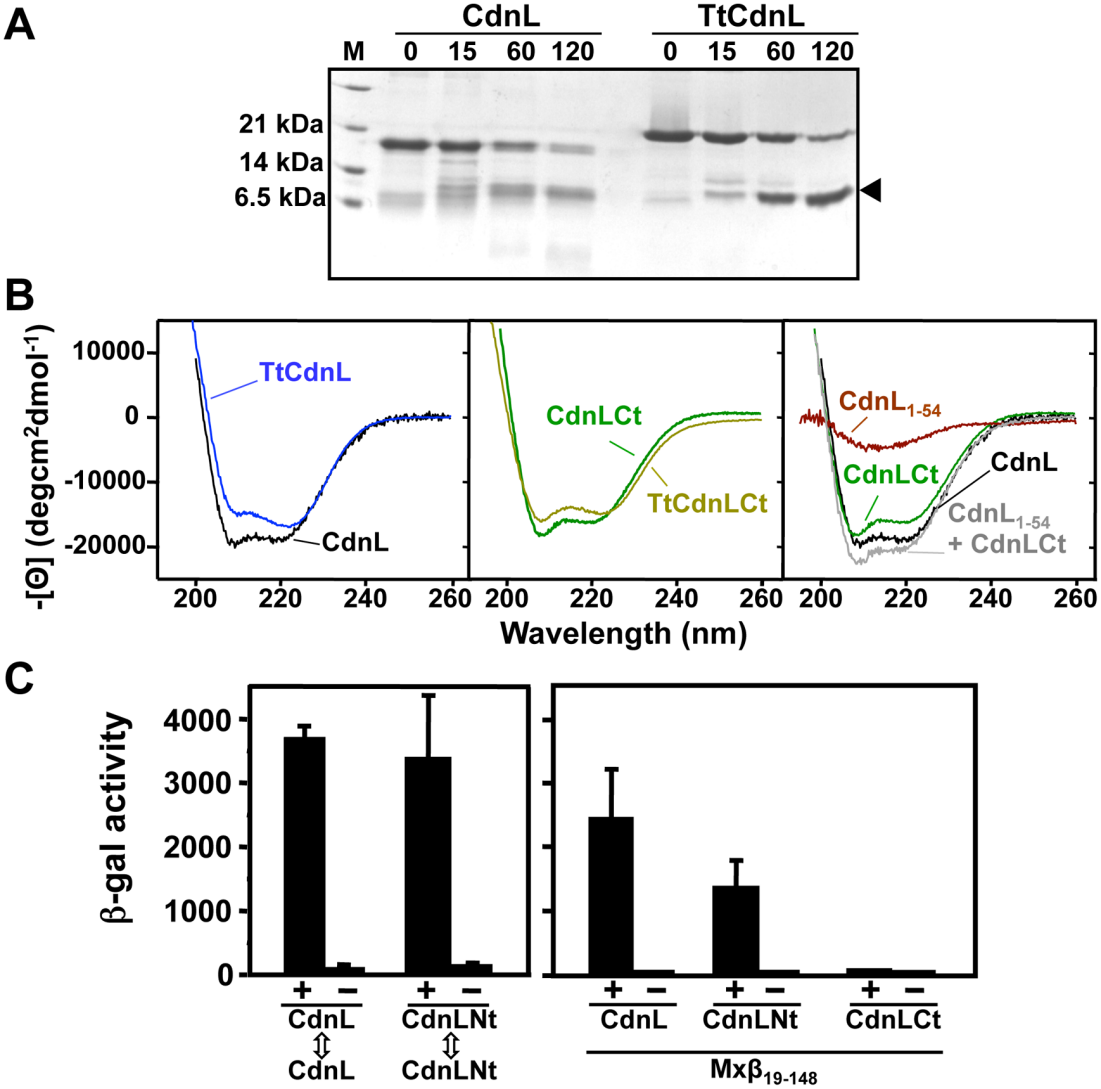
CdnL domain analysis. (**A**) Limited proteolysis of CdnL and TtCdnL. Aliquots of the reaction mix (1∶100 w/w subtilisin:protein) at 30°C were withdrawn at the times (in min) indicated on top and analyzed in Coomassie-stained 15% SDS-PAGE gels (lane “M”: molecular weight markers). Arrowhead on the right points to the subtilisin-resistant fragment, which mass spectrometry and N-terminal sequencing identified as the ∼100-residue C-terminal region in both proteins. Note the slower mobility of TtCdnL, whose size (164 residues) is the same as CdnL. (**B**) Far UV-CD spectra of CdnL, TtCdnL, and their indicated fragments. (**C**) BACTH analysis of the interactions of CdnL and its domains showing reporter *lacZ* expression in *E. coli* transformed with plasmids pKT25 and pUT18 bearing *cdnL* or the gene for CdnLNt (left panel); or with a pUT18C construct of the gene for Mxβ_19–148_ and pKT25 with *cdnL* or its indicated fragments (right panel). pKT25 without insert was used in negative controls (“−”).

CdnL, TtCdnL, and CarDNt sequences predict similar secondary structures with an ∼60-residue N-terminal part with five β-strands and a short helix, and an ∼100-residue C-terminal part with five helices (Figure S1 in File S1). Limited proteolysis of CdnL or TtCdnL yielded an ∼12 kDa stable fragment identified as their ∼110-residue C-terminal segments by mass spectrometry and N-terminal sequencing (Figure 1A). The protease susceptibility of the N-terminal regions therefore suggests that this part is likely to be more exposed and/or flexible in CdnL and TtCdnL. By comparison, our earlier data suggested that CarD is more protease-susceptible and no stable domain equivalent to that in CdnL or TtCdnL was observed [7]. Hence, CdnL and CarD may adopt different structures. Helix contents (in %) estimated from [Θ]_222_, the mean residue ellipticity at 222 nm in degcm^2^ dmol^−1^, in far-UV CD spectra (Figure 1B, left and middle panel) are comparable for CdnL (50%; [Θ]_222_ = −18,200) and TtCdnL (45%; [Θ]_222 = _−16,500), and for CdnLCt (43%; [Θ]_222_ = −15,700) and TtCdnLCt (38%; [Θ]_222_ = −13,800). Moreover, the full-length CdnL CD spectrum coincides well with the sum of those for CdnLCt and CdnL_1–54_, the remaining protease-sensitive CdnL N-terminal stretch from residue 1 to 54 that has the shallow β-sheet far-UV CD minimum at 215 nm (Figure 1B, right panel). Limited proteolysis and far-UV CD data thus indicate similar secondary structures for CdnL and TtCdnL, and that the predicted β-strand and α-helical segments segregate as protease-susceptible N-terminal and protease-resistant C-terminal domains, respectively.

Significant NMR signal broadening and overlap had suggested distinct CdnL forms in exchange and analytical ultracentrifugation indicated co-existing CdnL monomers and dimers in solution [24], [25]. CdnL self-interaction is also observed in BACTH analysis (Figure 1C). Since this behaviour complicates NMR analyses we examined the isolated CdnL domains. The excellent signal dispersion and overall quality of its NMR spectra allowed complete NMR chemical shift assignments for CdnLCt, which was monomeric as assessed by amide ^1^H T_2_ relaxation data or size-exclusion analysis [25]. CdnL_1–54_ was, however, unsuitable given its poor expression and NMR spectral quality, but a longer 68-residue CdnL N-terminal fragment, CdnLNt, yielded well-dispersed NMR spectra for which chemical shifts were completely assigned [24]. An apparent molecular weight from size-exclusion analysis of 12±1 kDa that was greater than the calculated 7.8 kDa monomer value suggested that CdnLNt self-interacts and this was confirmed by BACTH analysis (Figure 1C, left panel). Moroever, Mxβ_19–148_, the *M. xanthus* RNAP-β segment from residue 19 to 148, shown previously to interact with CdnL [4], does so with CdnLNt but not CdnLCt (Figure 1C, right panel). CdnLNt therefore appears to be the domain that mediates CdnL interactions with itself and with RNAP-β.

### Three-dimensional NMR solution structures of CdnL and its domains

**Figure 2:**
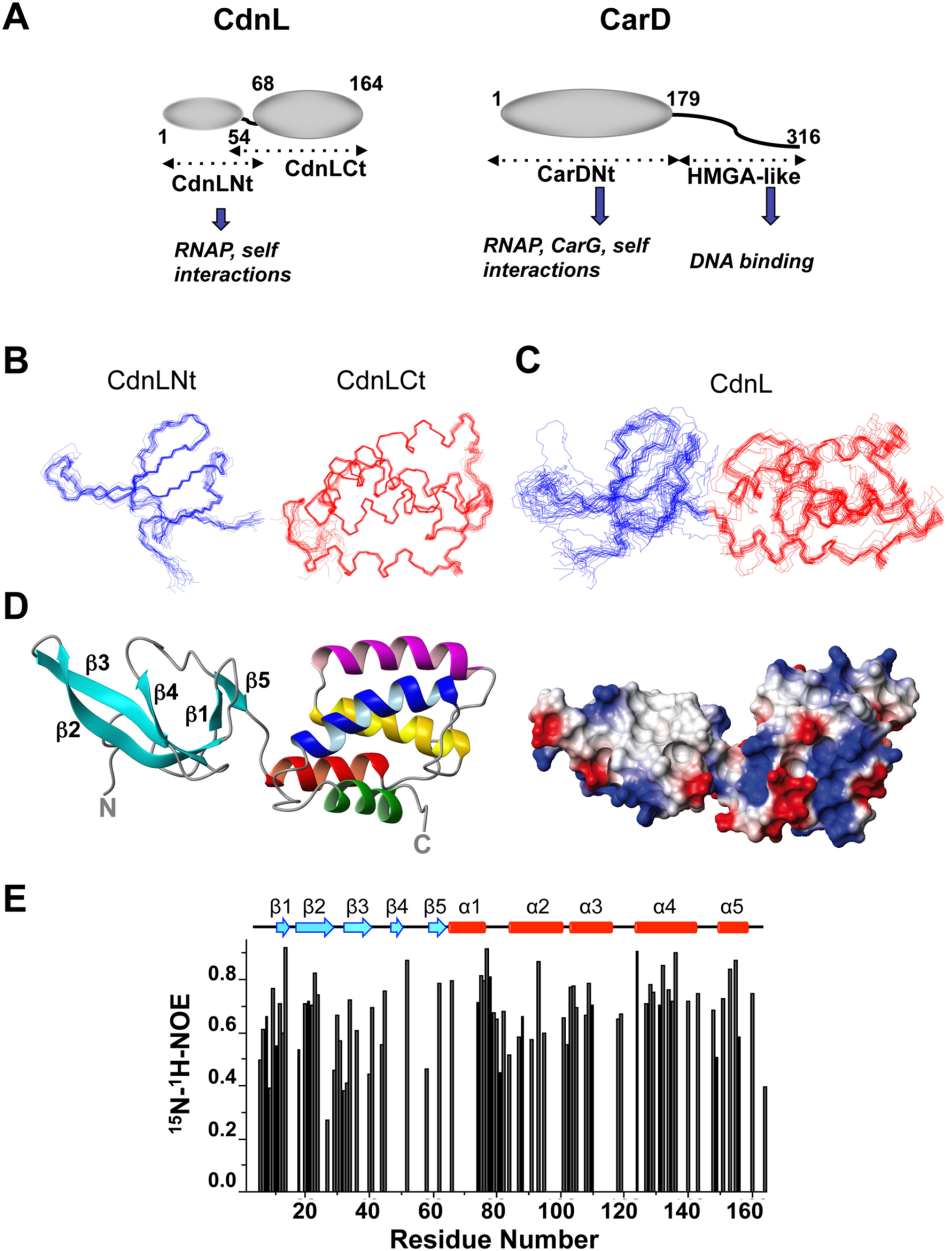
NMR solution structures of CdnL and its domains. (**A**) Schematic comparing CdnL and CarD domain architectures. Structured domains are shown as ellipsoids, intrinsically unstructured domains as wavy lines, and known interactions of each domain are indicated. Residues delimiting the CdnL and CarD domains are numbered. Note that CdnL resembles CarDNt in size and sequence, and lacks the HMGA-like DNA binding domain that is present in CarD. NMR structures of CdnLNt, CdnLCt, and full-length CdnL were determined in this study. (**B**) Superposition of the backbone traces for the 20 final NMR structures for CdnLNt (blue), CdnLCt (red), as indicated. (**C**) Same as in (**B**) for full length CdnL. (**D**) Average NMR solution structure of native CdnL in ribbon (left) and electrostatic surface representations (right). In the ribbon structure, the five β-strands (in cyan) of the CdnLNt domain are indicated, and the CdnLCt α-helices are colored red (α1), magenta (α2), yellow (α3), blue (α4) and green (α5). (**E**) Steady state backbone ^15^N-^1^H NOE (from the ratio of cross-peak intensities with and without ^1^H saturation at pH 7.0 and 25°C) plotted versus residue number obtained for 1 mM^ 15^N, ^13^C-labeled CdnL. Errors for the heteronuclear NOE data were estimated to be ≤5% based on average noise levels in the NMR spectra.

Figure 2A summarizes schematically the known CdnL domains and interactions, and those of CarD for comparison. We determined the NMR structures of isolated CdnLNt or CdnLCt in a straightforward manner. These were then used in a domain-parsing approach to aid NMR analysis of full-length CdnL (complicated by peak broadening and overlap), given the correspondence between the [^1^H,^15^N]-HSQC NMR cross peaks and medium and long-range NOEs that could be unambiguously assigned for CdnL and those for CdnLNt and CdnLCt [24]. The CdnLNt and CdnLCt NMR structural ensembles are well-defined (Figure 2B), with the pair-wise root-mean-square deviations (rmsd) for the structurally ordered backbone segments being (0.8±0.3) Å in CdnLNt (residues 5–62) and (0.4±0.1) Å in CdnLCt (residues 60–163 of CdnL; Table S2 in File S1). However, the CdnL ensemble was less well defined (rmsd of 1.6±1.0 Å for residues 5–162), but improved if only the ordered residue segments 5–53 (rmsd = 0.7±0.2Å) or 57–162 (rmsd = 0.5±0.4 Å) were considered (Figure 2C). This could stem from an insufficient number of constraints in the structure calculations and/or from a dynamic orientation of the two domains relative to each other in the full-length protein (Table S2 in File S1). [^15^N-^1^H] heteronuclear NOEs can inform on the local backbone flexibility and are typically high (>0.6) in the more rigid regions but lower where they are dynamic. Based on these, the more rigid regions in CdnL coincide, as is usually the case, with the α-helical and β-strand regions, with the segments between them and at the N- and C-termini being flexible. Such NOEs, however, could not be determined for the region linking the two CdnL domains due to problems of spectral overlap (Figure 2E). But a dynamic orientation of the two domains in solution would accord with residues 54 to 56 at the junction between the two domains (and located at the subtilisin cleavage site) being the only ones without ordered (φ, ψ) angles, other than the first seven (including three remaining from the tag) N- and the last two C-terminal residues.

CdnLNt adopts a twisted β-sandwich structure with antiparallel β5-β1-β2-β3-β4 topology and a 3_10_-helix between β4 and β5 (Figure 2D). We could detect most of the expected β-sheet cross-strand H-bonds (donor-acceptor bond distances ≤2.4 Å, bond angles <35°), but not intermolecular NOEs between CdnLNt molecules. CdnLCt is compact with five α-helices (spanning residues 66–76, 85–98, 104–118, 126–146, and 151–160, numbered as in the native protein) and the sequentially adjacent helices are in an antiparallel arrangement (Figure 2D). Besides characteristic α-helical (i, i+3) and (i, i+4) H-bonds, several salt-bridge interactions could be discerned (E66–K70 in α1; R91–E94, E97–K100 in α2; E109-R112 in α3; K146–E150 in α4; D156–K159 in α5). Helix α3 is almost entirely buried with solvent accessible surface areas (ASA) ≤10% for residues 104, 107, 108, and 110–116, and 10–25% for residues 105, 106, 109, 117, with only residue 118 (ASA≈36%) being solvent accessible. One face of α3 packs against the α1–α2 helical hairpin and the other against α4–α5, the helical topology being sustained by interhelical hydrophobic clusters, salt-bridges (α2/α3: K98/E106; α3/α4: K118/E127, α4/α5: R135/E154) and side-chain H-bonds (α1/α5: Y73/D156; α2/α3: Y101/E106; α3/α4: Y115/R135). Conserved residues with nonpolar aliphatic sidechains, distributed along the sequence in a leucine zipper-type heptad pattern in the α3–α4 segment (noted first in the CarD sequence [7], [14]), actually participate in interhelical packing interactions to form a so-called internal leucine zipper within the folded domain [21]. A striking structural feature of the overall acidic CdnLCt (theoretical pI = 4.99) is a patch of solvent-exposed nonpolar residues (W88, Y92, M96, and F125, with sidechain ASA of 26, 42, 61, 82%, respectively) surrounded by basic residues.

**Figure 3:**
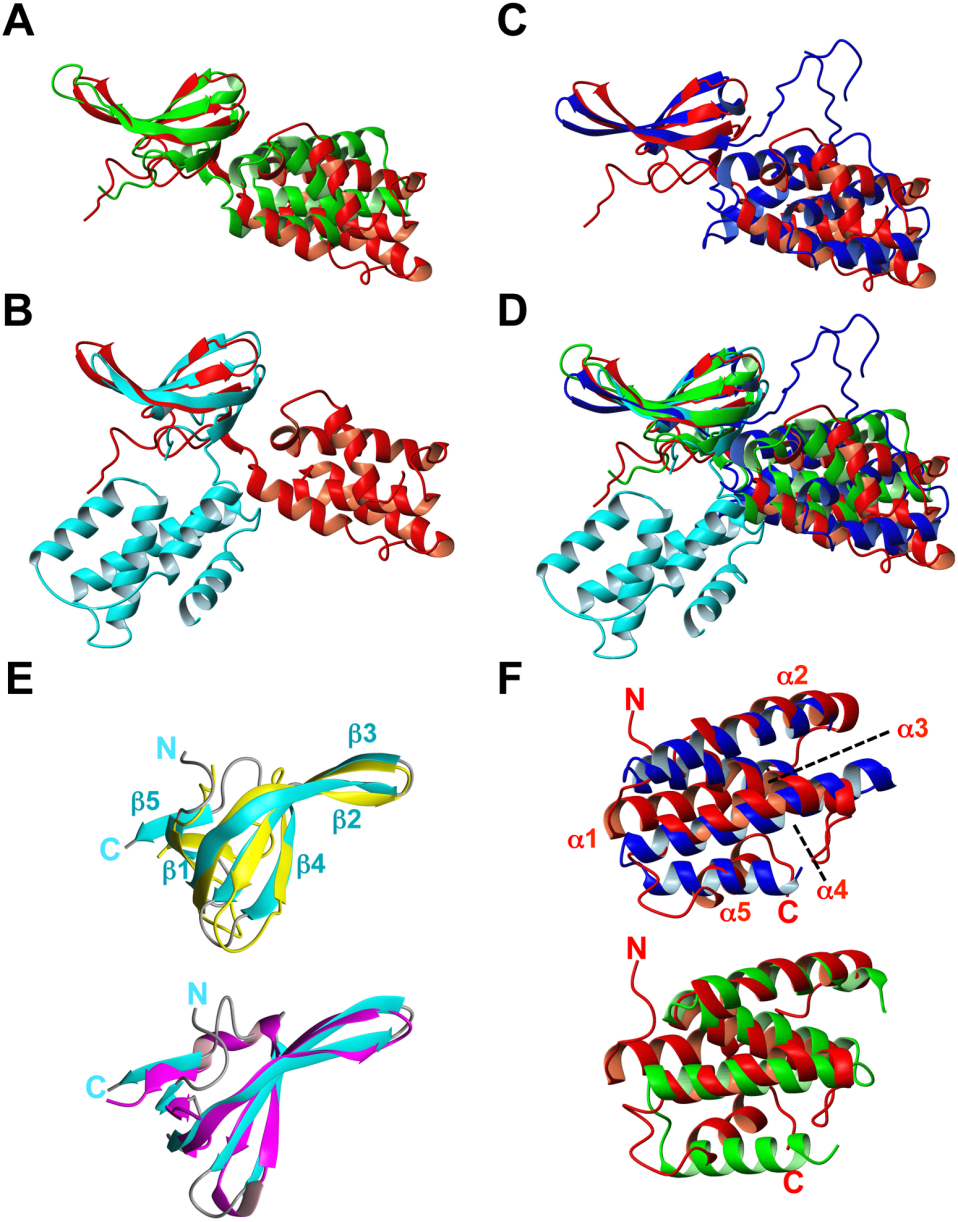
Structural comparisons with *M. xanthus* CdnL NMR structure. Backbone overlay of the CdnL NMR structure (red) onto the crystal structures of (**A**): TtCdnL (green; PDB: 4L5G); (**B**) one unit in the MtCdnL domain-swapped dimer (blue; PDB: 4ILU); (**C**) MtCdnL in complex with the RNAP β-lobe domain (cyan; PDB: 4KBM); (**D**) all four structures. The overlay shown was generated with optimal (maximum) superposition of the N-terminal domains of each structure. (**E**) Backbone overlay of CdnLNt (cyan; left) onto: (top) TtCdnLNt (gold; PDB ID: 2lqK); (bottom) *E. coli* TRCF-RID (magenta; PDB ID: 2eyq). (**F**) Backbone overlay of CdnLCt (red) onto the TPR domains of: (top) FK506-binding protein (blue; PDB ID: 1kt0); (bottom) CadC_pd_ (green; PDB ID: 3ly8). Structures were generated with MOLMOL.

Each isolated domain in our CdnL NMR solution structure closely resembles its counterpart in the crystal structures of TtCdnL [18], or of MtCdnL in a domain-swapped dimer [22] or in a 1∶1 complex with the RNAP-β lobe domain [21], reported while our manuscript was in preparation (Figure 3A–D; Table S3 in File S1). The CdnLNt NMR structure is also similar (rmsd 1.2 Å) to that of TtCdnLNt that we determined in parallel (Figure 3E; [20]). However, overall structures of the different CdnL homologs coincide less (rmsd ≥8 Å), even for the two reported MtCdnL forms, because the two domains in each structure adopt distinct relative orientations (Figure 3, Table S3 in File S1). This would be consistent with the two domains being flexibly linked, as we have inferred for *M. xanthus* CdnL and others have inferred for MtCdnL [21], [22], in contrast to the proposed fixed relative orientation of the two domains in TtCdnL [18]. Our observation that CdnL self-interacts via CdnLNt is echoed in the N-terminal β1 strand swap that produces a dimer of MtCdnL [22], which interestingly is a monomer in the complex with the RNAP-β lobe [21]. In all of the structures, the basic-nonpolar patch mentioned earlier stands out in the C-terminal domain. Besides these homologs, DALI structure database searches [36] matched the twisted β-sheet topology of CdnLNt to other Tudor-like domains [37], [38], the best hit being the 66-residue *E. coli* TRCF-RID (*Z*-score = 6.1; rmsd = 2.2 Å, 18% sequence identity; Figure 3E). A hit with a lower *Z-*score (3.4; rmsd = 2.4 Å) was TtTRCF-RID, whose reported structure has only the three central antiparallel β-strands well-defined [13]. Some bacterial proteins implicated in direct interactions with RNAP or in RNAP complexes like NusG and RapA (*Z-*scores 3.5–4.5) were also hits. Again, besides the hits to the CdnL homologs, DALI matched CdnLCt to the all-helical, C-terminal, tetratricopeptide repeat (TPR) module of the FK506-binding protein, FKBP51 (*Z*-score = 5.3, rmsd = 3.4 Å, 82 aligned residues, 12% sequence identity; [39]), and the most similar prokaryotic protein was CadC_pd_ (*Z*-score = 4.6, rmsd = 5.8 Å, 74 aligned residues, 5% sequence identity), the *E. coli* transcriptional activator CadC pH-sensory periplasmic domain [40]. CdnLCt helices α2–α4 superimpose onto α2–α4 of the six-helix FKBP51 TPR domain and α4–α6 of the CadC_pd_ 11-helix bundle (Figure 3F). TPR domains typically mediate protein-protein interactions and correspond to 3–16 tandem repeats (examples with only one or two repeats, or with those that are dispersed and not tandem have also been reported) of an ∼34-residue structural motif of degenerate sequence that folds as two antiparallel α-helices, of length 12 to 15 residues, crossing each other at an ∼24° angle and linked by a turn [41]–[43]. CdnL structure thus reveals two domains resembling known protein-protein interaction modules: a Tudor-like N-terminal and a TPR-like C-terminal domain.

### CdnL recognizes RNAP-β via conserved contacts

NMR chemical shift perturbations on titrating [^15^N, ^13^C] CdnL, CdnLNt, or CdnLCt with unlabeled Mxβ_19–148_ could reveal the contacting residues in the labeled proteins. However, the broad signals and poor dispersion in the Mxβ_19–148_ NMR spectra made it unsuitable for analysis. In qualitative accord with BACTH data that CdnL interacts with Mxβ_19–148_ via CdnLNt, the [^15^N, ^13^C] and [^1^H, ^15^N]-HSQC crosspeaks of [^15^N,^13^C] CdnL and CdnLNt, but not [^15^N, ^13^C] CdnLCt, broadened or disappeared on titrating with Mxβ_19–148_. These, however, failed to identify the contact residues.

**Figure 4:**
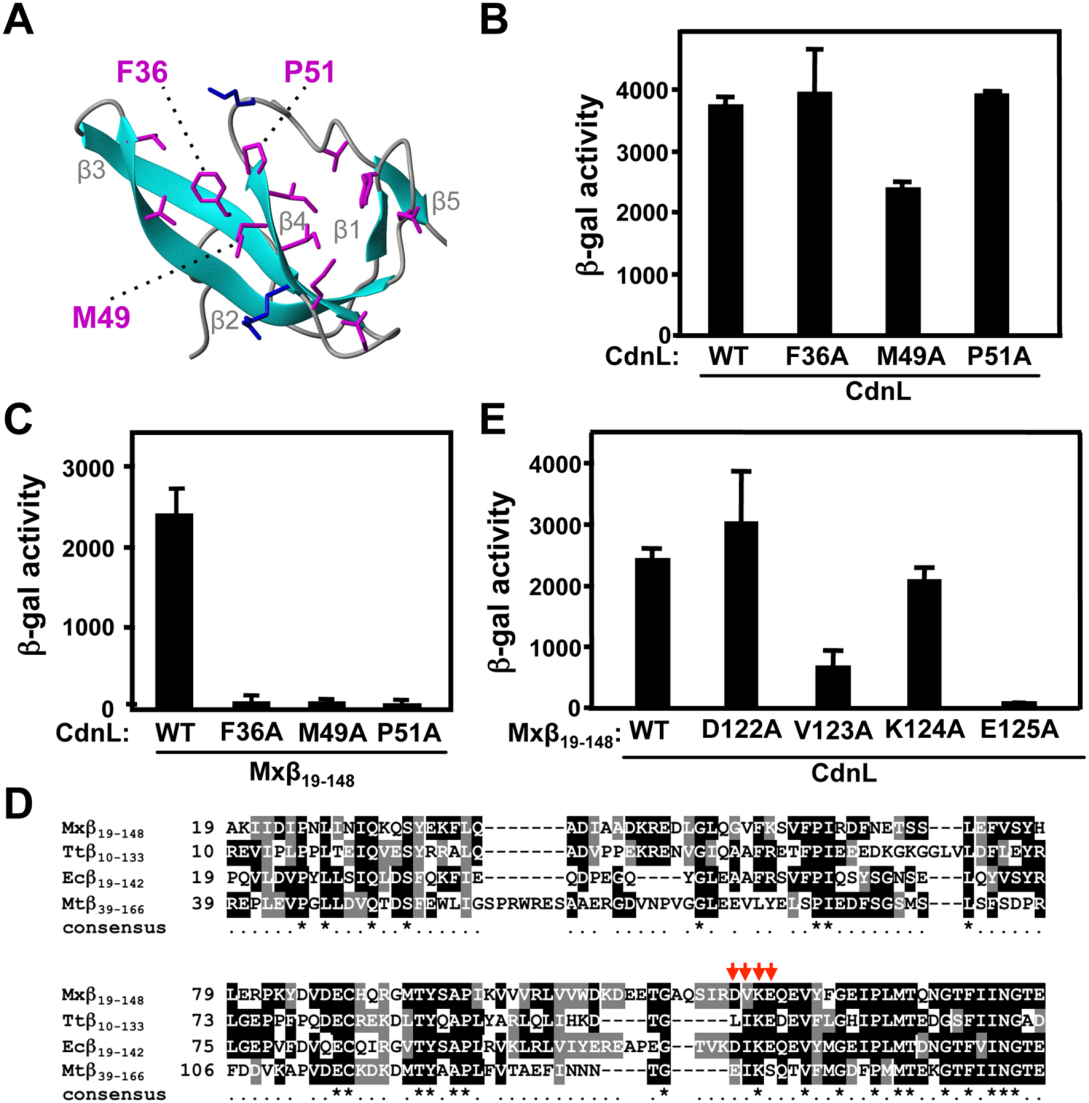
Mutational analysis of CdnL interaction with RNAP-β. (**A**) Native CdnLNt structure showing residue side chains that may contact RNAP-β, with the three labeled tested by mutational analysis (see text). (**B**) BACTH analysis showing that the F36A, M49A and P51A CdnL variants self-interact in cells with the pKT25 and pUT18 constructs of wild type or mutant *cdnL*, as indicated. (**C**) BACTH analysis of the interaction with Mxβ_19–148_ (in pUT18C) of CdnL F36A, M49A, or P51A mutants in pKT25. (**D**) Sequence alignment of the *M. xanthus* RNAP-β segment, Mxβ_19–148_ (NCBI code, YP_631280.1) and its equivalents in *T. thermophilus*, Ttβ_10–133_ (WP_014630291), in *E. coli,* Ecβ_19–142_ (P0A8V2), and in *M. tuberculosis* (CAB09390). Residues shaded black if identical, or grey if similar, in at least two of the aligned sequences. An asterisk in the consensus line below indicates conservation in all four sequences. Red arrows point to RNAP-β residues analyzed by site-directed mutagenesis in this study. (**E**) BACTH analysis of the interaction of Mxβ_19–148_ (WT) or its indicated mutants in pUT18C versus wild-type CdnL in pKT25.

We therefore tested how specific mutations in CdnL affected the interaction with Mxβ_19–148_. CdnL residues F36, M49, and P51 (Figure 4A) were chosen, since they align (Figure S2 in File S1) with three that contact RNAP-β in the structurally similar TtTRCF-RID (Y350, Y362, P364; [13]), TtCdnLNt (Y38, Y51, P53; [20]), and MtCdnL (Y34, R47, P49; [21]). All three are solvent-exposed in the native CdnL structure (ASA≥28%) and burial of their nonpolar side chains would energetically favor complex formation. In BACTH analysis, the CdnL mutants continued to self-interact (Figure 4B) indicating that they were stably expressed and properly folded. However, all three mutants were deficient in the interaction with Mxβ_19–148_ (Figure 4C). We also tested if the RNAP-β residues involved in contacts with CdnL mirror those observed for its homologs or TRCF-RID. For this, we mutated to A each residue in the *M. xanthus* RNAP-β segment D122-V123-K124-E125 (Figure 4D), which is quite conserved in various RNAP-β and has been implicated in such contacts in other studies [10], [13], [20], [21], [23], [44]. BACTH analysis (Figure 4E) indicated that mutating V123 or E125, but not D122 or K124, significantly impaired interaction with CdnL. By comparison, mutating L107, I108, or E110 (but not K109) in TtRNAP-β [20], or I147, K148, S149 in MtRNAP-β [23] impaired interaction with the cognate CdnL. Thus, other than species-specific variation in some contacts, cognate CdnL-RNAP-β pairs interact via conserved segments and surfaces on both proteins.

### Loss of CdnL-RNAP interaction impairs *M. xanthus* viability

**Figure 5:**
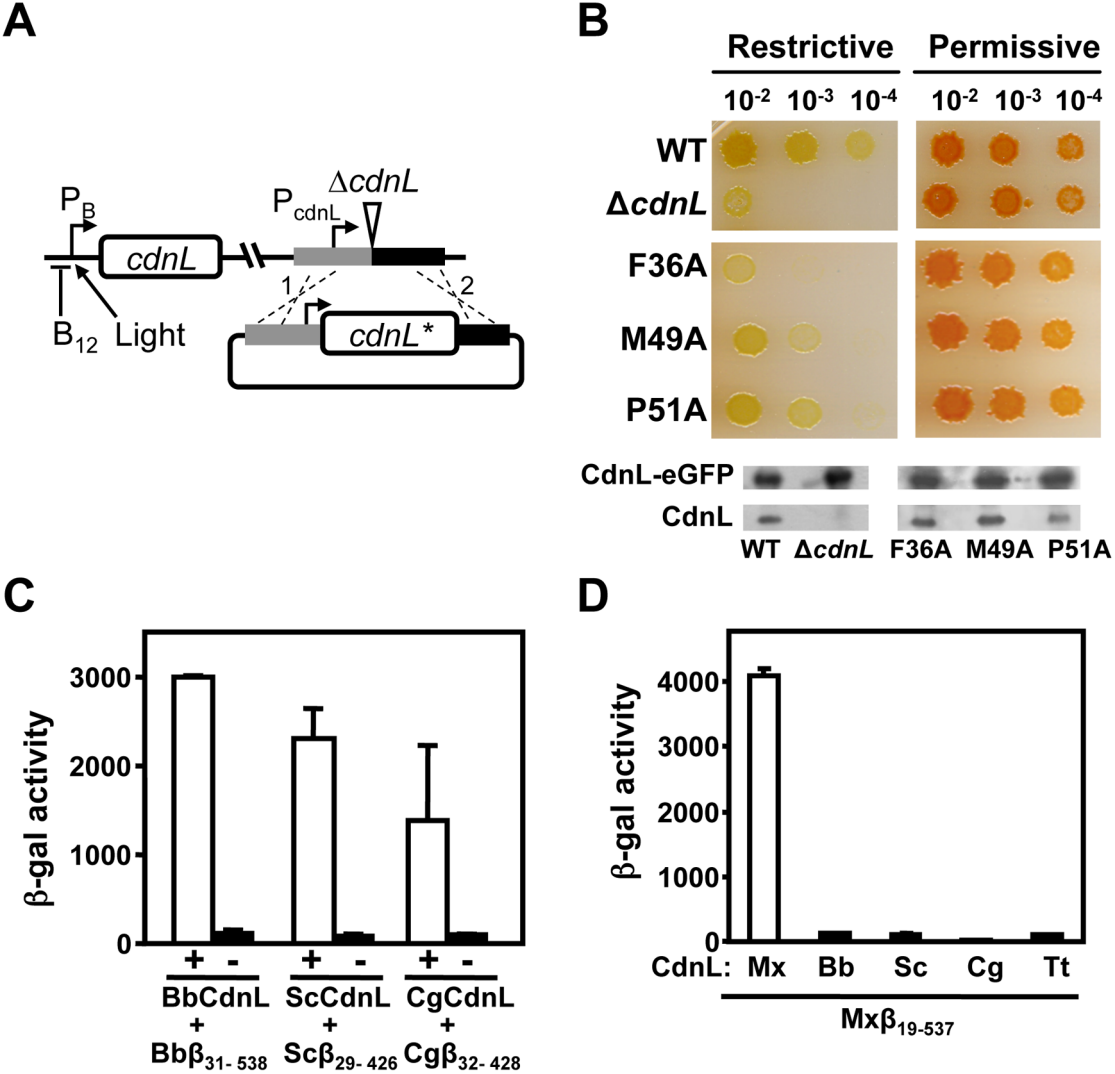
Consequences of disrupting CdnL interaction with RNAP *in* *vivo*. (**A**) Scheme of the strategy employed to check for *cdnL* complementation in *M. xanthus.* A pMR2873 construct with the required *cdnL* allele (cdnL*) under P_cdnL_ control and DNA segments flanking *cdnL* upstream (grey) and downstream (black) in the genome, was introduced into strain MR1467, which bears the Δ*cdnL* allele and a copy of *cdnL* at a heterologous site under the control of the photoinducible P_B_ promoter (expressed in the light but repressed in the presence of B_12_ in the dark). Merodiploids resulting from plasmid integration by recombination at either “1” or “2” would constitutively express cdnL* from P_cdnL_ and conditionally express the wild-type allele from P_B_ at a heterologous site. (**B**) Complementation analyses in *M. xanthus* with the F36A, M49A and P51A CdnL mutants. CTT/B_12_ plates that were spotted with 5 µl of liquid cultures (OD_550_ ∼1) grown under permissive conditions and diluted as indicated, and then incubated for 2 days at 33°C under restrictive (dark and B_12_) or permissive (light, hence the red color) conditions. “WT” is the positive control derived from using pMR2873 with wild-type *cdnL*, and “Δ*cdnL*” is the recipient strain MR1467. Western blots (using polyclonal anti-CdnL antibodies) of *M. xanthus* cell extracts from strains expressing each CdnL variant and in which CdnL-eGFP supplied the essential CdnL function, as described in the text. (**C**) BACTH analysis of the interaction of CdnL homologs (BbCdnL, ScCdnL, and CgCdnL) with Bbβ_31–538_, Scβ_29–426_, and Cgβ_32–428_ fragments, respectively. “+” below the open bars indicates that both members of the pair are present, while “−” is the negative control (without the indicated CdnL homolog). We have shown the interaction of TtCdnL with its cognate β-fragment elsewhere [18]. (**D**) BACTH analysis of the interaction of BbCdnL, ScCdnL, CgCdnL or TtCdnL with *M. xanthus* Mxβ_19–537_.

Next we tested if disrupting the interaction with RNAP affected the vital CdnL function in *M. xanthus*. For this, we resorted to the same strategy employed previously to check functional complementation of CdnL [4]. Briefly, a plasmid bearing *cdnL* or its mutant allele and flanking chromosomal segments was electroporated into the *M. xanthus* strain MR1467, which harbors the Δ*cdnL* allele at the native chromosomal locus and can express *cdnL* conditionally at a heterologous site from the photoinducible P_B_ promoter, which is downregulated by B_12_ (Figure 5A). Transformants resulting from chromosomal integration of the plasmid by homologous recombination at the endogenous *cdnL* locus were obtained under permissive conditions (i.e., growth in the light, which causes cells that are normally yellow in the dark to turn red due to light-induced carotenoid synthesis [4]). Under restrictive conditions (dark and B_12_ present), growth was impaired for cells expressing only the F36A, M49A, or P51A CdnL form, with F36A producing a phenotype as drastic as the *cdnL* deletion (Figure 5B). That this was not due to the CdnL mutants being unstable in *M. xanthus* was verified in Western blots of strains expressing each mutant and the essential CdnL function supplied by CdnL-eGFP [4]. The latter can be distinguished from the mutants using anti-CdnL antibodies due to its larger size and served as both a positive and loading control (Figure 5B, bottom). In MtCdnL, although mutating only R47 (equivalent to CdnL M49; Figure S2 in File S1) did not compromise *M. tuberculosis* viability, mutating it together with R25 (T27 in CdnL) impaired the interaction with RNAP and was lethal [23]. Thus, interaction with RNAP is critical for CdnL function. This prompted us to check if lack of interaction with *M. xanthus* RNAP-β could underlie the inability of CdnL homologs such as TtCdnL, BbCdnL (from *Bdellovibrio bacteriovorus*), CgCdnL (*Corynebacterium glutamicum*), or ScCdnL (*Streptomyces coelicolor*) to function in *M. xanthus* that we observed previously [4]. These homologs share with CdnL sequence identities ranging from 28% (for TtCdnL) to 60% (for BbCdnL) and conserve many of the residues reported to contact RNAP-β, including those tested here in CdnL (Figure S2A in File S1). While BbCdnL is from a δ-proteobacterium like *M. xanthus*, CgCdnL and ScCdnL are both actinobacteria like MtCdnL. We found that each of these homologs interacted with cognate RNAP-β using BACTH analysis (note that the longer β-lobe fragment had to be used in these cases, as has been reported with MtCdnL [23]), but none did so with Mxβ_19–537_ (Figure 5C), thereby providing an explanation for why these homologs failed to function in *M. xanthus*. Overall, the above results indicate that although the CdnL-RNAP interaction involves conserved surfaces and contacts, and is preserved in bacteria, it can nonetheless be species-specific.

### CdnLCt has a specific and crucial role in CdnL function

**Figure 6:**
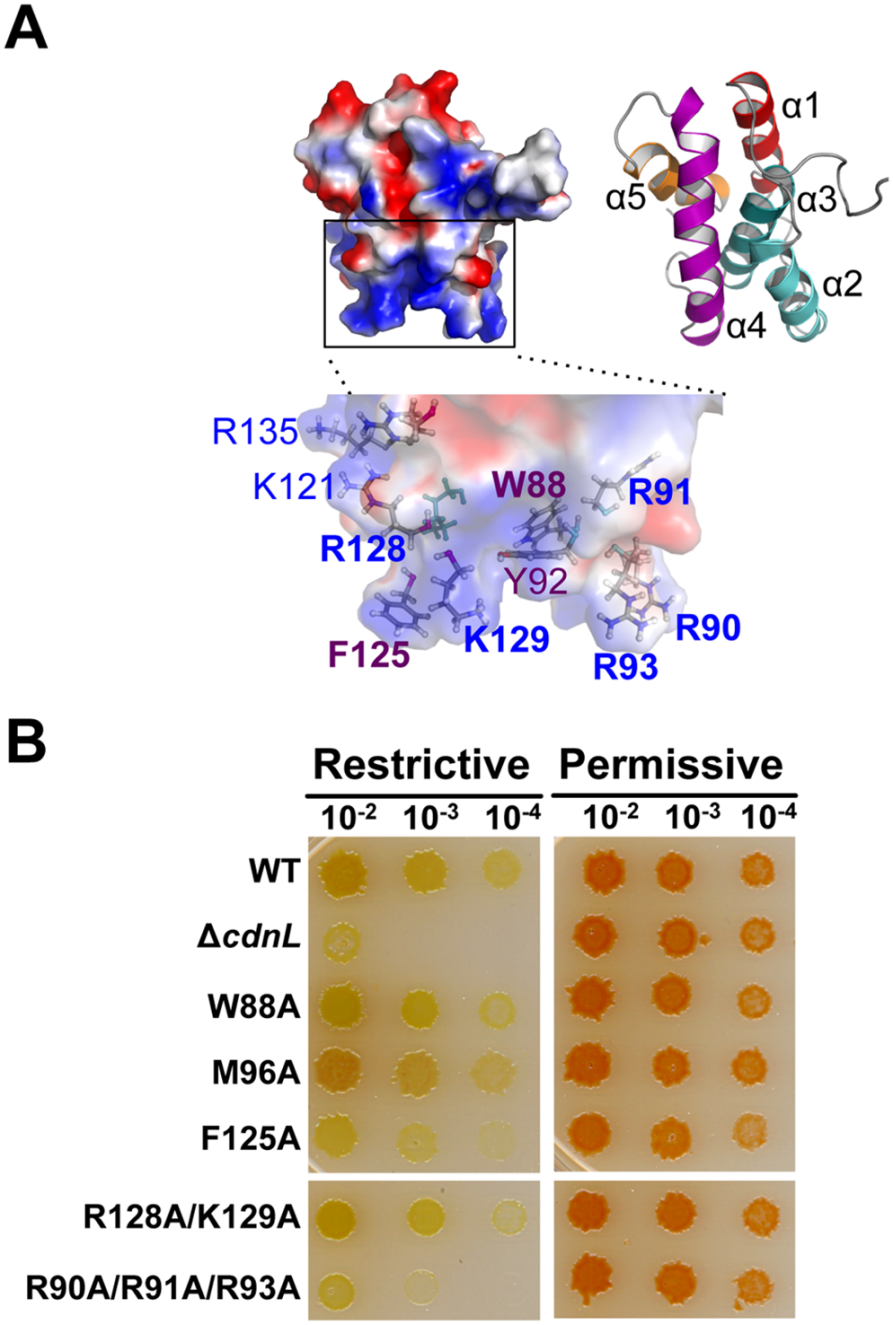
Consequences *in* *vivo* of mutating the CdnLCt solvent-exposed basic-hydrophobic patch. (**A**) CdnLCt electrostatic surface (left) and ribbon representations (right; each helix is labeled and colored differently) generated using PyMOL. The zoom shows sidechains (as sticks) of the labeled nonpolar and basic residues composing the solvent-exposed basic-hydrophobic patch. (**B**) Complementation analyses in *M. xanthus* of the CdnL W88A, M96A, F125A, R128A/K129A, and R90A/R91A/R93A mutants versus wild-type CdnL (WT) and Δ*cdnL*, as in Figure 5B.

Interaction with RNAP, while essential, does not suffice for CdnL function. In *M. xanthus*, CarDNt interacts with the endogenous RNAP yet it cannot functionally replace CdnL [4], nor can a CdnL variant that retains the RNAP-interacting segment but whose C-terminal domain has been swapped for the structurally similar one in TtCdnL (Figure S3A in File S1). This therefore points to the C-terminal part also having a crucial and specific role. Its structural similarity to TPR modules suggests that this could be in a protein-protein interaction, but no partner has thus far been identified for this domain of CdnL, although interestingly its counterpart in CarD interacts with CarG ([15], [16]; our unpublished data). Hence, to gain further insights into CdnLCt function we turned to structural clues, focussing on the residues comprising the solvent-exposed nonpolar-basic patch that stands out in the overall acidic CdnLCt (Figure 6A) and are also conserved in CdnL homologs (Figure S2 in File S1; Table S4 in File S1; [18], [21]). A basic patch hints at DNA binding but our previous study suggested that CdnL lacks this ability [4]. In EMSA analysis, neither CdnL nor its domains exhibited DNA binding even at high protein concentrations (10–38 µM), regardless of probe length, or the presence or absence of nonspecific competitor DNA (Figure S3B, C in File S1). It is worth noting that CarDNt also did not bind DNA, unlike full-length CarD, which has been repeatedly shown to exhibit minor groove DNA binding to AT-rich tracts via its HMGA-like module [7], [15], [16], [27]. On the other hand, MtCdnL has been reported to exhibit weak, nonspecific DNA binding [18], [21]. CdnL thus appears to differ from its homolog in being unable to bind DNA.

We next probed if mutating specific residues in the basic-hydrophobic patch affected CdnL function *in* *vivo*. Thus, we mutated to A the nonpolar W88 (highly conserved), M96 (less conserved), and F125 (least conserved but most solvent-exposed), or simultaneously two (R128/K129) or three (R90/R91/R93) basic residues in the patch. BACTH analysis confirmed that all these CdnL mutants continued to interact with Mxβ_19–148,_ as expected, and Western blots (as in Figure 5B) showed that they were stably expressed in *M. xanthus* (Figure S3D in File S1). When tested in *M. xanthus*, only the F125A and the R90A/R91A/R93A mutants showed a noticeable growth defect under restrictive conditions, the triple mutant being almost as adversely affected as the Δ*cdnL* strain (Figure 6B) or the CdnL mutants deficient in RNAP interactions (Figure 5B). Mutations of corresponding basic residues in MtCdnL have been reported to reduce its DNA binding *in* *vitro*, with that equivalent to R90A/R91A/R93A producing the greatest effect [21], but their consequences *in* *vivo* have not been described. It is thus noteworthy that mutating the basic R90/R91/R93, but not R128/K129, is detrimental to function *in* *vivo* despite no apparent DNA binding by *M. xanthus* CdnL. Additionally, the lack of an effect on mutating the highly conserved W88 in *M. xanthus* CdnL contrasts with the observation that the equivalent mutation in MtCdnL or TtCdnL impairs function [18], [21]. Altogether, these data reveal that a stretch of basic residues in the CdnL C-terminal domain is crucial for its essential function in *M. xanthus*.

### CdnL stabilizes RNAP-binding and transcription from σ^A^-dependent promoters

**Figure 7:**
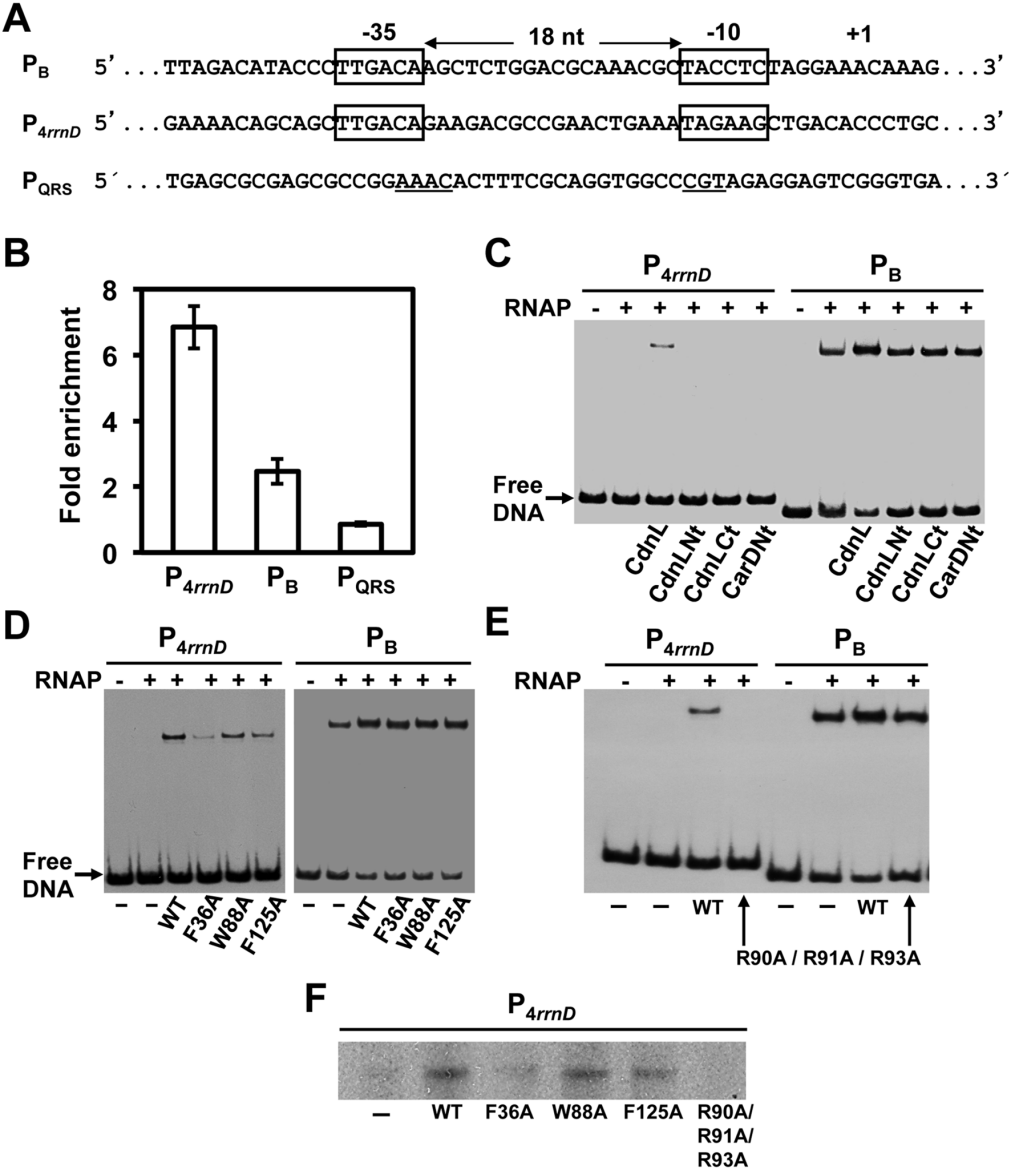
CdnL favors RP_o_ formation and transcription from σ^A^-dependent promoters. (**A**) Promoter sequences for the σ^A^-dependent P_4*rrnD*_ and P_B_, indicating the -35 and -10 elements (in boxes) and the transcription start site. For comparison, the ECF-σ CarQ-dependent P_QRS_ promoter is also included, with the conserved motifs at the -35 and -10 regions underlined. (**B**) ChIP-qPCR analysis of CdnL enrichment at P_4*rrnD*_, P_B_ and P_QRS_ in *M. xanthus* carried out as described in the text. The mean and standard of three independent experiments are shown. (**C**)–(**E**) EMSA of the binding of the ^32^P-labeled 130-bp P_B_ or 151-bp P_4*rrnD*_ DNA probes to 130 nM RNAP-σ^A^ holoenzyme, alone or in the presence of 10 µM of CdnL, CdnLNt, CdnLCt, or CarDNt (**C**), or a given CdnL variant (**D, E**), as indicated. After incubation for 30-min at 37°C, the complexes formed were challenged by adding 1 µg heparin. Note that any alteration in the migration of the shifted complexes due to CdnL binding to RNAP was not discernible, possibly because CdnL is considerably smaller than RNAP. (**F**) Single round, run-off *in* *vitro* transcription from P_4*rrnD*_ (see Materials and methods) with 130 nM RNAP-σ^A^ alone or with 10 µM CdnL (WT or mutant) followed by heparin challenge (1 µg) and then addition of the labeled NTP mix to initiate transcription. In **C**–**F**, representative data from three or more experiments are shown.

First described as a repressor of rRNA transcription [5], mycobacterial CdnL has now been reported to be an activator of rRNA promoters that stimulates formation of the transcriptionally competent open complex (RP_o_), and its colocalization *in* *vivo* with the major housekeeping σ (σ^A^) at promoter regions has led to the proposal that it may function as a global regulator of transcription initiation [18]. Such a role for CdnL has, however, remained untested in almost all other bacteria where it exists including *M. xanthus*. Interestingly, *M. xanthus* CarD has no apparent effect on rRNA promoters but is required for the action of several ECF-σ factors at their target promoters [17]. We therefore tested if CdnL is linked to the activity of *M. xanthus* promoters requiring σ^A^, such as the P_4*rrnD*_ rRNA promoter [17], [45] and the light-inducible P_B_ [28], which have identical -35 elements (matching the σ^A^ promoter consensus) but different -10 elements, separated in both promoters by a 18-bp spacer. Chromatin immunoprecipitation (ChIP) was used to probe if CdnL associates with P_4*rrnD*_ and P_B_ *in* *vivo* (to ensure expression of P_B_, the experiments were performed in a *carR* mutant background [46]; Table S1 in File S1). Relative to an intragenic control region, CdnL was enriched at P_4*rrnD*_ and also, albeit at lower levels, at P_B_. By contrast, no association of CdnL was observed with P_QRS,_ which does not depend on σ^A^ and CdnL, but rather on the ECF-σ factor CarQ and on CarD (Figure 7A, B; [17]).

In EMSA with P_4*rrnD*_ or P_B_ as DNA probes, RNAP-σ^A^ holoenzyme yielded a retarded band that appeared to be more intense if CdnL was also present (Figure S3C in File S1). Similar results were observed for P_B_ on challenge with heparin, which dissociates nonspecific and closed RNAP-σ^A^-promoter complexes but leaves RP_o_ (compare Figure 7C with Figure S3C in File S1). With the P_4*rrnD*_ probe and RNAP-σ^A^, no retarded band was detected in the presence of heparin (RP_o_ complexes at rRNA promoters disocciate rapidly and have short lives [2]), but the band appeared if CdnL was present prior to addition of heparin (compare Figure 7C with Figure S3C in File S1). This effect was not observed with isolated CdnL domains, or CarDNt, indicating that only full-length CdnL is capable of stabilizing RP_o_ formation (Figure 7C). This was further tested with the two loss-of-function CdnL mutants that phenocopy a Δ*cdnL* strain (F36A that does not interact with RNAP, and R90A/R91A/R93A that does interact), and two other CdnL mutants that interact with RNAP and cause mild or no apparent effects *in* *vivo* (F125A and W88A, respectively). Differences among CdnL and its mutants in stabilizing RP_o_ were subtle at P_B_, but more pronounced at P_4*rrnD*_ (Figure 7D, E): the stable RP_o_ complex at P_4*rrnD*_ continued to be detected with the W88A or F125A mutants, was faint with F36A, and disappeared with R90A/R91A/R93A. RP_o_ formation and stability is a crucial rate-limiting step in transcription especially at rRNA promoters [2], and the effects due to the presence of CdnL or each of its above mutants in heparin-challenged, single round transcription run-off assays from P_4*rrnD*_ *in* *vitro* (Figure 7F) correlated well with the EMSA results on stable RP_o_ formation. That disrupting the interaction with RNAP precludes CdnL from stabilizing RP_o_ formation at an rRNA promoter has also been observed with MtCdnL, but the wild-type behaviour of the W88A CdnL mutant contrasts with the defect in RP_o_ stabilization and transcription observed for the equivalent mutant of MtCdnL [18]. Additionally, and for the first time, our data reveal that the R90A/R91A/R93A mutant, despite interacting with RNAP, fails to stabilize RP_o_ formation, notably at P_4*rrnD*_. Moreover, CdnL mutants defective in RP_o_ stabilization are also the ones detrimental to cell growth and viability.

## Discussion

The CdnL NMR solution structure and its structure-based mutational analysis in this study provide molecular insights into the cellular roles and modes of action of this RNAP-binding protein that is essential for growth and viability but has unknown functions in *M. xanthus*. CdnL has a two-domain architecture in solution. Its N-terminal protease-susceptible domain recognizes RNAP-β and adopts a twisted β-sheet Tudor domain-like fold very similar to those found in the TRCF RNAP interacting domain, in the TtCdnL N-terminal domain NMR structure that we determined in parallel [20], and in crystal structures of full-length TtCdnL [18] and MtCdnL [21], [22] reported while this manuscript was under preparation. The second CdnL module is a compact, protease-resistant C-terminal domain with an α-helical fold that is conserved in its homologs and resembles the TPR protein-protein interaction domains of some proteins. An interacting partner for the *M. xanthus* CdnL C-terminal domain has not been identified thus far but, interestingly, its counterpart in CarDNt (whose structure remains to be determined) does mediate a protein-protein interaction: that with CarG (our unpublished data). A solvent-exposed patch of nonpolar residues surrounded by basic ones is conspicuous in the acidic CdnL C-terminal domain and is conserved in its homologs. We found that a set of these basic residues is crucial for CdnL function.

The NMR solution structures of the CdnL N- and C-terminal domains closely match those of its homologs in crystal, but the relative orientations of the two domains vary considerably in the distinct structures, even between the two crystal structures reported for MtCdnL. A plausible explanation is that the two domains are flexibly linked, as inferred for CdnL in this study and for MtCdnL elsewhere [21], [22], rather than rigidly maintained as proposed in TtCdnL [18]. A conformationally flexible N-terminal domain can rationalize its protease sensitivity in CdnL or TtCdnL, and the N-terminal β1-strand swap to produce the MtCdnL dimer [22], which appears as a monomer in the complex with the RNAP-β lobe [21]. Our data also show that CdnL self-interacts via its N-terminal domain and exists as monomers and dimers in solution [24], [25]. CdnL may therefore be inherently flexible with domain motions that could be functionally relevant.

Interaction with RNAP is indispensable for CdnL function in *M. xanthus*, since a single mutation disrupting it (e.g. F36A) was sufficient to impair viability, as in mycobacteria [23], the only other species where CdnL has been studied *in* *vivo*. The CdnL-RNAP interaction involves conserved surfaces and contacts, and we verified in this study that various other CdnL homologs also interact with their cognate RNAP. However, none of these homologs recognized *M. xanthus* RNAP and they failed to function in this myxobacterium [4]. Thus, the CdnL-RNAP interaction, while conserved, appears to be also species-specific. Ability to interact with RNAP is essential but not sufficient for function, and crucial and specific determinants also reside in the C-terminal domain. Thus, CdnL function in *M. xanthus* cannot be replaced by CarDNt, which also interacts with cognate RNAP [4], nor by a variant that retains the RNAP-interacting part of CdnL but whose C-terminal module is swapped for the structurally equivalent one from TtCdnL. Moreover, our analysis revealed for the first time that also critical for CdnL function *in* *vivo* is a basic R90-R91-R93 stretch in the C-terminal domain, which is part of a basic-nonpolar surface patch mentioned earlier. Mutating R90-R91-R93 did not affect interaction with RNAP but produced a lethal, loss-of-function phenotype similar to a Δ*cdnL* strain, whereas mutating the also basic R128–K129 in this patch had no apparent effect. Both basic segments are conserved in MtCdnL (in TtCdnL, R90 is A and K129 a Q) and mutating either impaired the intrinsically weak, non-specific DNA-binding of MtCdnL although their effects on cell growth and viability were unreported [21]. *M. xanthus* CdnL, however, lacks intrinsic DNA-binding ability and the drastic phenotype produced by mutating R90-R91-R93, but not the proximal R128–K129, suggests a specific and crucial role for R90-R91-R93 in CdnL function.

In *M. xanthus* CdnL appears to localize at σ^A^-dependent promoters *in* *vivo* and stabilizes formation of transcriptionally competent RP_o_ complexes *in* *vitro*, most notably at rRNA promoters, as was also observed in mycobacteria [5], [18]. Whether promoters other than those requiring σ^A^ can be targeted by CdnL is not yet known, to our knowledge. At least at one ECF-σ-dependent promoter tested, we could not discern a preferential association of CdnL *in* *vivo*. The underlying molecular details on how CdnL acts at σ^A^-dependent promoters remain to be elucidated. Based on the structure of TtCdnL, a model has been proposed in which its RNAP-binding via the N-terminal module positions the basic C-terminal patch and the conserved W for interaction with promoter DNA around its −12 region in the upstream fork of the transcription bubble [18]. In contrast to MtCdnL or TtCdnL, which depend on the conserved W for function [18], mutating this W in CdnL did not impair function *in* *vivo* or RP_o_ stabilization *in* *vitro* suggesting that the mechanistic details may vary in *M. xanthus*. In the above structural model, the two TtCdnL domains are in a fixed relative orientation and no significant protein-protein interactions between TtCdnL and σ^A^ in RP_o_ are predicted. The relative orientation of the two domains may, however, be flexible in CdnL (this study) and in MtCdnL [21], [22]. Moreover, in another structural model based on its dimer structure, docking one MtCdnL monomer unit onto RNAP-bound σ^A^ reportedly caused the other dimer unit to be proximal and so capable of interacting with σ^A^ [22]. Such an interaction could rationalize the action of CdnL at σ^A^ promoters. Although we could not detect any direct interaction of CdnL with σ^A^ or its isolated domains using BACTH analysis (our unpublished data), the possibility remains that this may occur in the context of the RP_o_ complex.

Stabilization of RP_o_ at σ^A^ promoters by CdnL would represent a distinct facet of the mechanisms employed to ensure transcription initiation by specific σ-associated RNAP, and related strategies appear to be employed by some other bacterial transcription factors that do not bind DNA. Examples include RbpA, a small, RNAP-binding protein found exclusively in actinobacteria, which is essential in mycobacteria but dispensable in *Streptomyces* [47]–[49], and Crl, known thus far only in γ-proteobacteria, where it is not vital [50]. RbpA and Crl show no sequence similarity, yet both have been reported to bind to RNAP core as well as to domain σ_2_ in their respective σ partners (σ^A^ and the closely related stress-induced σ^B^ for RbpA; σ^S^ for Crl), leading to the suggestion that they have analogous roles in transcription initiation [47]–[50]. RbpA stimulates transcription from, among others, rRNA promoters and, as mentioned above, is essential in mycobacteria. It is therefore intriguing that it co-exists with MtCdnL, which appears to carry out a similar task at rRNA promoters and is also essential. As with MtCdnL, mechanistic aspects of how RbpA and Crl promote RNAP assembly and later steps in transcription initiation remain to be addressed. The dual interaction of RbpA or Crl with RNAP and σ has been proposed to act as a bridge that could facilitate RNAP holoenzyme assembly and later steps in transcription including promoter binding and open complex formation and stability [47]–[50]. Such a scenario is also plausible for CdnL, with its N-terminal domain tethering it to RNAP-β and a flexibly linked C-terminal domain providing additional crucial contacts with specific elements of the RP_o_ complex. Future high-resolution studies of CdnL in the RP_o_ complex can provide molecular details on these and their mechanistic implications.

### Accession Numbers

Accession codes for CdnLNt, CdnLCt, and CdnL are, respectively, 2LT4, 2LT3 and 2LWJ for the structural coordinates deposited in the Protein Data Bank; and 15977, 18121 and 18151 for NMR chemical shifts in BioMagResBank (http://www.bmrb.wisc.edu/).

## Supporting Information

File S1 **Contains Tables S1–S4, Figures S1–S3 and legends, and References.**
(PDF)
