# Supplementary material for: Structural Insights into RNA Polymerase Recognition and Essential Function of Myxococcus xanthus CdnL
Source: PLoS One. 2014 Oct 1;9(10):e108946. doi: 10.1371/journal.pone.0108946 (PMC4182748; doi:10.1371/journal.pone.0108946)
Supplement: File S1 — Contains Tables S1–S4, Figures S1–S3 and legends, and References. (PDF) [file pone.0108946.s001.pdf]

SUPPORTING INFORMATION

Structural insights into RNA polymerase recognition and essential function of *Myxococcus xanthus* CdnL

Aránzazu Gallego-García[1], Yasmina Mirassou[2], Diana García-Moreno[1], Montserrat Elías-Arnanz[1]*, María Angeles Jiménez[2]*, and S. Padmanabhan[2]*

[1] Departamento de Genética y Microbiología, Área de Genética (Unidad Asociada al IQFR-CSIC), Facultad de Biología, Universidad de Murcia, Murcia, Spain

[2] Instituto de Química Física 'Rocasolano' (IQFR), Consejo Superior de Investigaciones Científicas (CSIC), Madrid, Spain

*Address correspondence to Montserrat Elías Arnanz (E-mail: melias@um.es), María Angeles Jiménez (E-mail: majimenez@iqfr.csic.es), or to S. Padmanabhan (E-mail: padhu@iqfr.csic.es).

Table S1. *Myxococcus xanthus* strains and plasmids used in this work

***M. xanthus* strain**	**Description**	**Source / Reference**
MR151	*carR*-inactivated (*carR3*) strain with light-independent expression of P_B and P_{QRS}	(1)
MR1467	Δ*cdnL* P_B::*cdnL* P_C::*carH* Δ*carA* Δ*carH* (XbaI site replaces endogenous *cdnL*). Tc^R	(2)
MR1482	MR1467 derivative with *cdnL* at the endogenous site. Tc^RKm^R	(2)
MR1488	*cdnL* at the endogenous site; P_{cdnL}::*cdnL-eGFP* at a heterologous site. Tc^R	(2)
MR1489	Δ*cdnL* at the endogenous site; P_{cdnL}::*cdnL-eGFP* at a heterologous site. Tc^R	(2)
MR1978	MR1467 with gene for CdnL(1-54)-TtCdnLCt. $Tc^RKm^RGal^S$	This work
MR2404	MR1467 with *cdnL(F36A)*. $Tc^RKm^RGal^S$	This work
MR2405	MR1467 with *cdnL(M49A)*. $Tc^RKm^RGal^S$	This work
MR2406	MR1467 with *cdnL(P51A)*. $Tc^RKm^RGal^S$	This work
MR2407	MR1489 with *cdnL(F36A)*. $Tc^RKm^RGal^S$	This work
MR2408	MR1489 with *cdnL(M49A)*. $Tc^RKm^RGal^S$	This work
MR2409	MR1489 with *cdnL(P51A)*. $Tc^RKm^RGal^S$	This work
MR2413	MR1489 with *cdnL(F125A)*. $Tc^RKm^RGal^S$	This work
MR2414	MR1489 with gene for CdnL(1-54)-TtCdnLCt. $Tc^RKm^RGal^S$	This work
MR2415	MR1467 with *cdnL(F125A)*. $Tc^RKm^RGal^S$	This work
MR2417	MR1467 with *cdnL(W88A)*. $Tc^RKm^RGal^S$	This work
MR2418	MR1467 with *cdnL(M96A)*. $Tc^RKm^RGal^S$	This work
MR2419	MR1467 with *cdnL(R128A/K129A)*. $Tc^RKm^RGal^S$	This work
MR2420	MR1467 with *cdnL(R90A/R91A/R93A)*. $Tc^RKm^RGal^S$	This work
MR2422	MR1489 with *cdnL(W88A)*. $Tc^RKm^RGal^S$	This work
MR2423	MR1489 with *cdnL(M96A)*. $Tc^RKm^RGal^S$	This work
MR2424	MR1489 with *cdnL(R128A/K129A)*. $Tc^RKm^RGal^S$	This work
MR2425	MR1489 with *cdnL(R90A/R91A/R93A)*. $Tc^RKm^RGal^S$	This work
**Plasmid**	**Description**	**Source / Reference**
pET15b	Vector for overexpressing proteins with an N-terminal His_6 tag. Amp^R	Novagen
pKT25	Vector for C-terminal fusion constructs to the T25 fragment of CyaA for use in bacterial two-hybrid analysis. Km^R	(3)
pTYB12	Vector for overexpressing proteins with an N-terminal intein tag. Amp^R	NE Biolabs
pUT18	Vector for N-terminal fusion constructs to the T18 fragment of CyaA for bacterial two-hybrid analysis. Amp^R	(3)
pUT18C	Vector for C-terminal fusion constructs to the T18 fragment of CyaA for bacterial two-hybrid analysis. Amp^R	(3)
pMR2873	Vector to insert a gene of interest at the endogenous *cdnL* site for complementation analysis. Km^RGal^S	(2)
pMR2914	pET15b-*cdnL*	(2)
pMR2973	pKT25-*cdnL*	(2)
pMR3040	pET15b-*TtcdnL*	This work
pMR3070	pMR2873-*cdnL*	(2)
pMR3207	pET15b construct to overexpress CdnLCt	(4)
pMR3260	pET15b construct to overexpress $CdnL_{1-54}$	This work
pMR3265	pUT18-*cdnL*	This work
pMR3318	pET15b construct to overexpress TtCdnLCt	This work
pMR3331	pMR2873 with coding sequence for the CdnL(1-54)-TtCdnLCt chimera	This work
pMR3407	pKT25-*TtcdnL*	This work
pMR3409	pKT25 with gene for CdnLNt	This work
pMR3410	pKT25 with gene for *M. xanthus* $TRCF_{514-645}$	(2)
pMR3412	pUT18C with gene for $Mx\beta_{19-148}$	(2)

Plasmid	Description	Source / Reference
pMR3429	pTYB12 construct to overexpress Mx$\beta_{19\text{-}148}$	This work
pMR3510	pKT25 with *C. glutamicum cdnL*	This work
pMR3511	pKT25 with *S. coelicolor cdnL*	This work
pMR3512	pKT25 with *B. bacteriovorus cdnL*	This work
pMR3520	pTYB12 construct to overexpress CdnLNt	This work
pMR3585	pUT18C with gene for $\beta_{19\text{-}148}$(D122A)	This work
pMR3586	pUT18C with gene for $\beta_{19\text{-}148}$(V123A)	This work
pMR3587	pUT18C with gene for $\beta_{19\text{-}148}$(K124A)	This work
pMR3588	pUT18C with gene for $\beta_{19\text{-}148}$(E125A)	This work
pMR3616	pKT25 with gene for CdnLCt	This work
pMR3619	pUT18C with gene for CdnLNt	This work
pMR3622	pKT25-*cdnL(M49A)*	This work
pMR3623	pKT25-*cdnL(P51A)*	This work
pMR3624	pKT25-*cdnL(F36A)*	This work
pMR3671	pMR2873-*cdnL(F36A)*	This work
pMR3672	pMR2873-*cdnL(M49A)*	This work
pMR3673	pMR2873-*cdnL(P51A)*	This work
pMR3711	pUT18-*cdnL(F36A)*	This work
pMR3712	pUT18-*cdnL(M49A)*	This work
pMR3713	pUT18-*cdnL(P51A)*	This work
pMR3727	pUT18C with gene for *B. bacteriovorus* $\beta_{31\text{-}548}$	This work
pMR3728	pUT18C with gene for *S. coelicolor* $\beta_{29\text{-}426}$	This work
pMR3729	pUT18C with gene for *C. glutamicum* $\beta_{32\text{-}428}$	This work
pMR3730	pUT18C with gene for *M. xanthus* $\beta_{19\text{-}537}$	This work
pMR3879	pUT18C with gene for *T. thermophilus* $\beta_{10\text{-}395}$	This work
pMR3819	pET15b contruct to overexpress CdnL(F36A)	This work
pMR4053	pMR2873-*cdnL(F125A)*	This work
pMR4068	pKT25-*cdnL(F125A)*	This work
pMR4093	pMR2873-*cdnL(M96A)*	This work
pMR4095	pMR2873-*cdnL(W88A)*	This work
pMR4108	pKT25-*cdnL(W88A)*	This work
pMR4110	pKT25-*cdnL(M96A)*	This work
pMR4131	pET15b contruct to overexpress CdnL(W88A)	This work
pMR4133	pET15b contruct to overexpress CdnL(F125A)	This work
pMR4159	pMR2873-*cdnL(R128A/K129A*	This work
pMR4160	pMR2873-*cdnL(R90A/R91A/R93A)*	This work
pMR4180	pET15b to overexpress CdnL(R90A/R91A/R93A)	This work
pMR4181	pKT25-*cdnL(R128A/K129A)*	This work
pMR4182	pKT25-*cdnL(R90A/R91A/R93A)*	This work

Table S2. Structural statistics for the ensemble of the 20 lowest energy NMR structures

	CdnLNt[a]	CdnLCt[a]	CdnL[a]
**NOE distance constraints**			
Intraresidue (*i–j*=0)	211	570	839
Sequential (|*i–j*|=1)	76	331	503
Medium range (1<|*i–j*|<5)	59	543	696
Long-range (|*i–j*| ≥5)	148	644	1026
Total number	494	2088	3064
Averaged total per residue	7.2	18.5	20.3
**Dihedral angle constraints**			
φ angles	63	106	155
ψ angles	48	87	135
Total	111	193	290
**Maximum constraints violations**			
Distance (Å)	0.18±0.07	0.2±0.1	0.5±0.2 (0.5±0.2)[b]
Dihedral angle (º)	1.6 ± 0.2	1.5±0.4	3.8±1.6 (5.9±3.8)[b]
**Averaged structure energies**			
CYANA target function value	0.51±0.01	1.3±0.1	4.8±0.7 (7.1±1.3)[b]
AMBER energy (kcal/mol)	− 2656	− 4877	− 6745
Van der Waals energy (kcal/mol)	− 414	− 853	− 1179
Electrostatic energy (kcal/mol)	− 3952	− 7274	− 10320
**Deviations from ideal geometry**			
Bond length (Å)	0.014	0.013	0.013
Bond angle (º)	1.9	1.9	1.9
**Pairwise rmsd (Å) (Backbone/Heavy atoms)**			
All residues	2.9±1.0/3.7±1.0	0.8±0.2/1.2±0.2	1.7±1.0/2.1±0.9
Ordered residues	0.8±0.3/1.4±0.3	0.4±0.1/0.9±0.1	1.6±1.0/1.9±0.9<br>*0.7±0.2/1.3±0.2*[c]<br>*0.5±0.4/1.2±0.3*[d]
**Ramachandran analysis (ordered/all residues)**			
Most favoured regions (%)	80.5/76.6	89.1/88.8	87.1/85.4
Additional allowed regions (%)	18.6/22.5	10.5/10.8	12.5/14.0
Generously allowed regions (%)	0.9/0.9	0.4/0.4	0.4/0.6
Disallowed regions (%)	0.0/0.0	0.0/0.0	0.0/0.0

[a]PDB accession codes: (i) CdnLNt: 2LT4; (ii) CdnLCt: 2LT3; (iii) CdnL: 2LWJ;. Ordered residues (numbered from the N-terminus as in native CdnL): (i) CdnLNt: 5 to 62; (ii) CdnLCt: 60 to 163; (iii) CdnL: 5 to 53 and 57 to 162. The N-terminal cloning tag, AGH, was excluded from these analyses. [b]Values corresponding to the calculation performed including RDC's are given between parenthesis. The maximum averaged residual dipolar coupling (RDC) constraint violation is 0.7±0.5. [c]Values obtained considering only the ordered residues 5-53 are in italics. [d]Values obtained considering only the ordered residues 57-162 are in italics.

Table S3. Pairwise RMSD (in Å) for superposition of CdnL structures

		TtCdnL[a]		MtCdnL[b]		MtCdnL[c]
		A	B	A	B	
CdnL		1.7/3.6/5.1	1.6/3.6/5.2	1.7/3.1/8.7	2.0/3.1/8.7	1.2/2.1/7.7
TtCdnL[a]	A			2.2/2.6/10.7	2.0/2.5/10.8	1.8/2.9/7.0
	B			2.1/2.4/10.7	2.1/2.4/10.7	1.6/2.8/7.1
MtCdnL[b]	A				0.4/0.2/0.3	1.0/1.7/11.2
	B					1.1/1.6/11.1

PDB accession codes are as follows. CdnL: 2LWJ. [a] TtCdnL, 4L5G.A and 4L5G.B (crystallizes as two molecules in the asymmetric unit that were refined as two independent structures); [b] MtCdnL domain-swapped dimer: 4ILU and 4MFR; [c] MtCdnL in complex with RNAP-β lobe: 4KBM. RMSD is listed (in the order shown) considering the N-terminal domain β2-β3-β4 alone, the C-terminal domain α1-α2-α3-α4-α5 alone, or full-length protein. RMSD for the structures of full-length CdnL versus its homologs was estimated for optimal (maximum) superposition of the N-terminal domains in the structures.

Table S4. Conservation of mutated CdnLCt residues in the basic-hydrophobic patch

Taxonomical class	*M. xanthus* CdnL residue[a]							
	W88	M96	R90	R91	R93	F125	R128	K129
δ-Proteobacteria	W	M/L/S/Q	R/K/Q	R	R /K	F(Y,H)	R (K)	R (K)
CarD homologs	W	L/A	Q(V,I)	R	R	T/A	R	E
α-Proteobacteria	W	E/D	R	R	Q	Y/F	R	Q/E/T
Actinobacteria	W	L/Q/V/R	R	R	K	A/T/D	K,R	R,Q
Firmicutes	W	L/M/Q/A/I/T	V	R	R/N/H/Q	S/T/A/G	R/K	K/R/M
Deinococcus-Thermus	W/F	S/A/L/G/D	A	R	R	D/E/G/T/N/S	R/L/H	R/S/E/G/Q
Spirochaetales	W	L/M/V/E	Q	R	Q/T	L/I/V/A	K	R

[a]Residues within parentheses are found in few CdnL homologs; / is used if the residue on the right is also found in several CdnL homologs. CarD homologs, which occur only in δ-Proteobacteria, are listed separately within this group. Bacterial classes not shown (β-, γ-, and ε-proteobacteria, Cyanobacteria, Chlamydiae, Bacteroidetes) lack CdnL.

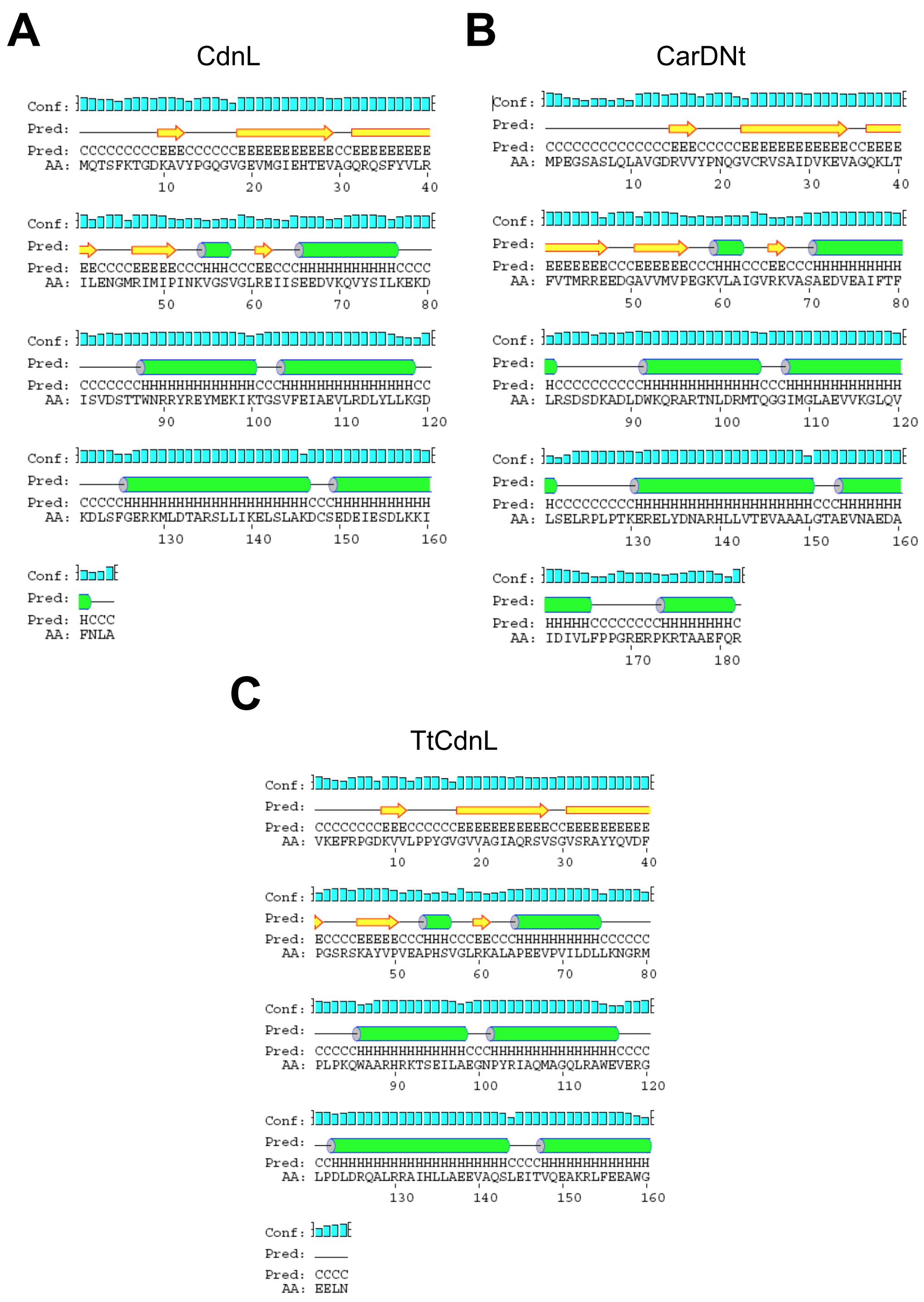

Figure S1. Sequence-based secondary structure prediction using PSIPRED (http://bioinf.cs.ucl.ac.uk/psipred/) for: (**A**) CdnL; (**B**) CarDNt; (**C**) TtCdnL. In the predictions, shown diagrammatically, AA is the query sequence with residue number below, Pred is the predicted secondary structure (unstructured: C/straight line; β-strand: E/yellow arrow; helix: H/green rod), and Conf is the confidence level (blue bar) of the prediction at each residue position.

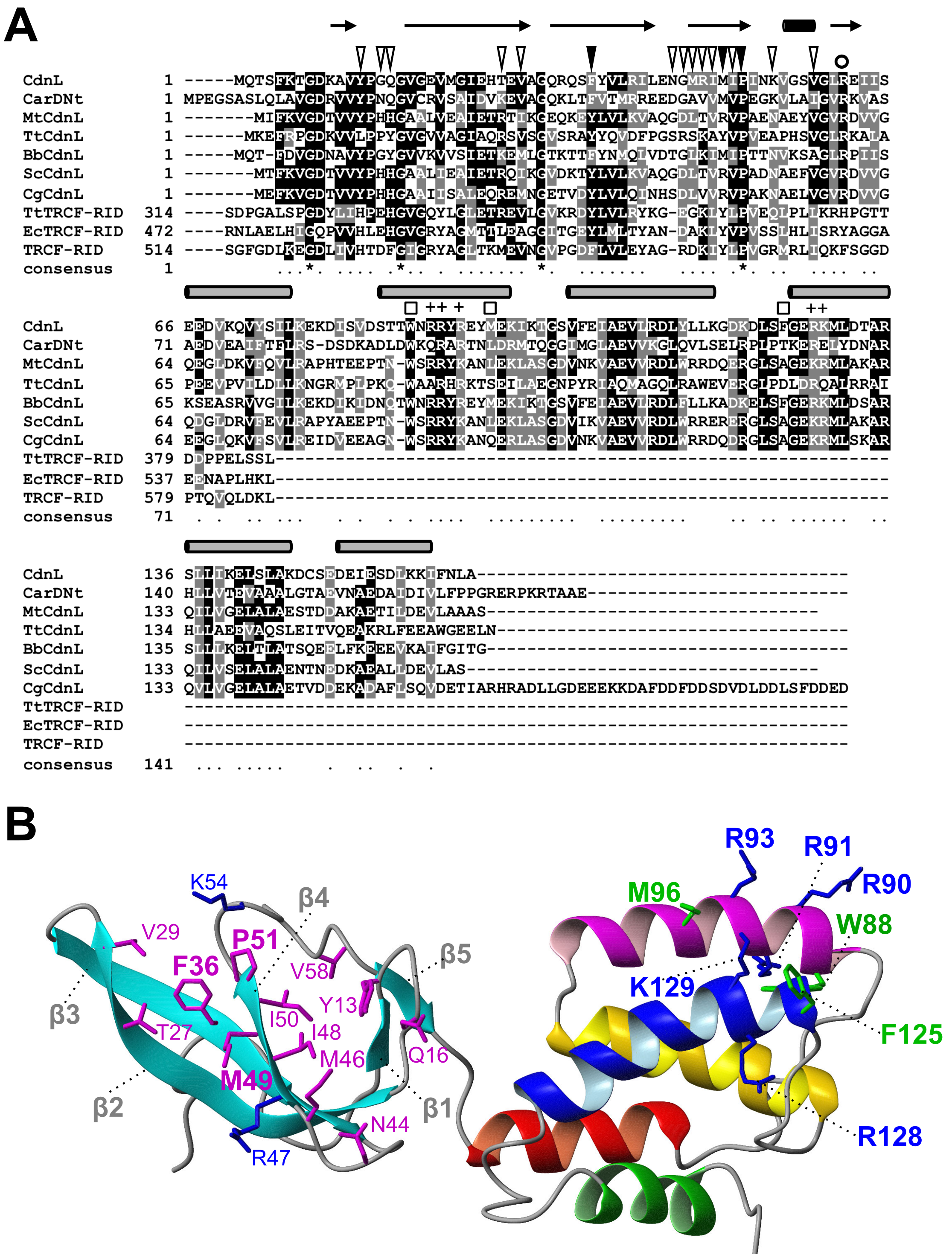

Figure S2. (**A**) Sequence alignment of CdnL, CarDNt, MtCdnL, TtCdnL, BbCdnL, ScCdnL, CgCdnL, TtTRCF-RID, EcTRCF-RID, and TRCF-RID (respective NCBI accession codes: YP_630846, CAA91224, CAA17859, YP_005787, NP_969149, NP_628406, YP_001139479, AAS80881, AAB26029, and YP_629274). Prefixes Mt, Tt, Bb, Sc, Cg, and Ec, respectively, indicate *M. tuberculosis*, *T. thermophilus*, *B. bacteriovorus*, *S. coelicolor*, *C. glutamicum*, and *E. coli* proteins, with no such prefix for the *M. xanthus* proteins. Residues are shaded black when identical in the majority of the aligned sequences, or grey when

similar. The asterisk in the consensus line below indicates that the residue above is identical in all of the sequences. Arrowheads point to RNAP-β contact residues reported for TtTRCF-RID or MtCdnL (6,7); filled arrowheads, open squares and "+" indicate CdnL and CarD residues probed using site-directed mutagenesis in this study; "o": the N-terminus of the subtilisin-resistant fragment. Secondary structural elements as determined from the CdnL NMR structure in this study are shown above the CdnL sequence with arrows for β-strands, grey rods for α-helices, and the short black rod for a 3_{10}-helix. (**B**) Native CdnL in ribbon representation displaying the side-chains of residues in the N-terminal domain that contact RNAP-β (see also (**A**)), and of residues that form the basic-hydrophobic patch in the C-terminal domain (see main text). Residues labeled in larger font and boldface were examined by site-directed mutagenesis in this study. The β-strands (in cyan) are labeled, and the five α-helices are colored in red (α1), magenta (α2), yellow (α3), blue (α4) and green (α5).

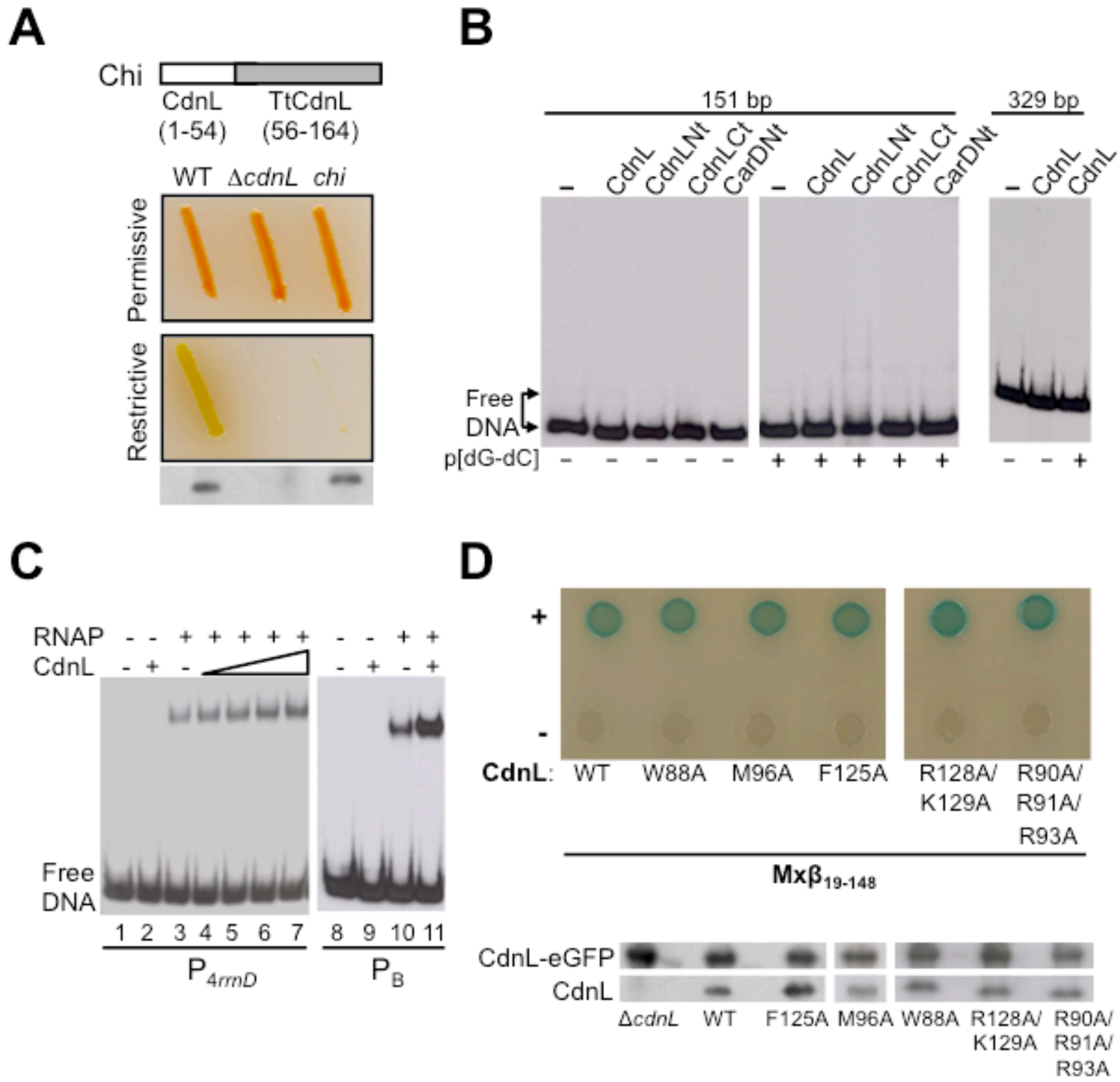

Figure S3. (**A**) Lack of function in *M. xanthus* shown by complementation analysis of Chi, the chimera in which the CdnL C-terminal domain is swapped for the structurally similar one from TtCdnL, represented schematically on top. Chi is stably expressed as inferred from Western blot analysis shown below. (**B**) EMSA showing that CdnL, its isolated domains, or CarDNt do not exhibit DNA binding *in vitro*. Reactions were performed with the P_{4rrnD} probe (151 bp or 329 bp, as indicated), 10 μM of protein and in the absence or presence of competitor poly[dG-dC]. Note that no DNA binding was observed even at CdnL concentrations as high as 38 μM. (**C**) Effect of increasing concentrations of CdnL (0.1, 1, 10 and 38 μM CdnL in lanes 4-7, respectively; left panel) on the binding of 130 nM RNAP to the 151 bp P_{4rrnD} probe or to the P_B probe (10 μM CdnL, lane 11; right panel) in the presence of poly[dG-dC] as nonspecific competitor and with no heparin. DNA probe alone (lanes 1, 8), or with only CdnL (38 μM, lane 2; 10 μM, lane 9), or with only 130 nM

RNAP (lanes 3 and 10) are also shown. Note that any alteration in the migration of the shifted complexes from CdnL binding to RNAP was not discernible, possibly because of the considerably smaller size of CdnL relative to RNAP. (**D**) On top is a spot assay on plates showing the interaction ("+"; blue spots) using BACTH analysis of the indicated CdnL mutants (in pKT25) with Mx$\beta_{19\text{-}148}$ (in pUT18C). As negative controls ("-"), pKT25 without insert was used. In the bottom are Western blots (using polyclonal anti-CdnL antibodies) of *M. xanthus* cell extracts from strains expressing each of the indicated CdnL variant and in which CdnL-eGFP supplied the essential CdnL function, as described in the main text.

REFERENCES

1. Balsalobre, J.M., Ruiz-Vázquez, R.M. and Murillo, F.J. (1987) Light induction of gene expression in *Myxococcus xanthus*. *Proc. Natl. Acad. Sci. U. S. A.*, **84**, 2359-2362.
2. García-Moreno, D., Abellón-Ruiz, J., García-Heras, F., Murillo, F.J., Padmanabhan, S. and Elías-Arnanz, M. (2010) CdnL, a member of the large CarD-like family of bacterial proteins, is vital for *Myxococcus xanthus* and differs functionally from the global transcriptional regulator CarD. *Nucleic Acids Res.*, **38**, 4586-4598.
3. Karimova, G., Ullmann, A. and Ladant, D. (2000) A bacterial two-hybrid system that exploits a cAMP signaling cascade in *Escherichia coli*. *Methods Enzymol.*, **328**, 59-73.
4. Mirassou, Y., García-Moreno, D., Santiveri, C.M., Santoro, J., Elías-Arnanz, M., Padmanabhan, S. and Jiménez, M.A. (2009) ^{1}H, ^{13}C and ^{15}N backbone and side chain resonance assignments of the C-terminal domain of CdnL from *Myxococcus xanthus*. *Biomol. NMR Assign.*, **3**, 9-12.
5. Gulten, G. and Sacchettini, J.C. (2013) Structure of the *Mtb* CarD/RNAP β-lobes complex reveals the molecular basis of interaction and presents a distinct DNA-Binding domain for *Mtb* CarD. *Structure*, **21**, 1859-1869.
6. Westblade, L.F., Campbell, E.A., Pukhrambam, C., Padovan, J.C., Nickels, B.E., Lamour, V. and Darst, S.A. (2010) Structural basis for the bacterial transcription-repair coupling factor/RNA polymerase interaction. *Nucleic Acids Res.*, **38**, 8357-8369.
7. Gallego-García, A., Mirassou, Y., Elias-Arnanz, M., Padmanabhan, S. and Jiménez, M.A. (2012) NMR structure note: N-terminal domain of *Thermus thermophilus* CdnL. *J. Biomol. NMR*, **53**, 355-363.
